# Validation of Network Communicability Metrics for the Analysis of Brain Structural Networks

**DOI:** 10.1371/journal.pone.0115503

**Published:** 2014-12-30

**Authors:** Jennifer Andreotti, Kay Jann, Lester Melie-Garcia, Stéphanie Giezendanner, Eugenio Abela, Roland Wiest, Thomas Dierks, Andrea Federspiel

**Affiliations:** 1 Department of Psychiatric Neurophysiology, University Hospital of Psychiatry, University of Bern, Bern, Switzerland; 2 Laboratory of Functional MRI Technology, Ahmanson-Lovelace Brain Mapping Center, Department of Neurology, University of California Los Angeles, Los Angeles California, United States of America; 3 Neuroimaging Research Laboratory (LREN), Department of Clinical Neurosciences, Vaud University Hospital Center (CHUV), Lausanne, Switzerland; 4 University Institute of Diagnostic and Interventional Neuroradiology, Inselspital and University of Bern, Bern, Switzerland; Universiteit Gent, Belgium

## Abstract

Computational network analysis provides new methods to analyze the brain's structural organization based on diffusion imaging tractography data. Networks are characterized by global and local metrics that have recently given promising insights into diagnosis and the further understanding of psychiatric and neurologic disorders. Most of these metrics are based on the idea that information in a network flows along the shortest paths. In contrast to this notion, communicability is a broader measure of connectivity which assumes that information could flow along all possible paths between two nodes. In our work, the features of network metrics related to communicability were explored for the first time in the healthy structural brain network. In addition, the sensitivity of such metrics was analysed using simulated lesions to specific nodes and network connections. Results showed advantages of communicability over conventional metrics in detecting densely connected nodes as well as subsets of nodes vulnerable to lesions. In addition, communicability centrality was shown to be widely affected by the lesions and the changes were negatively correlated with the distance from lesion site. In summary, our analysis suggests that communicability metrics that may provide an insight into the integrative properties of the structural brain network and that these metrics may be useful for the analysis of brain networks in the presence of lesions. Nevertheless, the interpretation of communicability is not straightforward; hence these metrics should be used as a supplement to the more standard connectivity network metrics.

## Introduction

Diffusion weighted imaging (DWI) together with tractography algorithms [1]–[6] provide a non-invasive method to localize and analyze white matter (WM) fiber tracts in-vivo and hence characterize the structure of physical connections in the connectome. More recently, methods of computational network analysis have been used to analyze the structural brain network topology at a large-scale and to investigate interactions between the different cortical regions [7]–[9]. Briefly, regions of interest (ROIs) defining the nodes of the network are given by a gray matter parcellation scheme and the weighted or binary connections (edges) between these nodes are defined using tractography. Several scalar metrics can then be computed to characterize and compare the complex topology of brain networks at the global and the local level [10]–[12]. This approach has recently been found to be a powerful tool to detect differences in the network topology specific to neurologic and psychiatric disorders [13]–[16].

The most commonly used network metrics in literature assume that information flowing between two regions will pass through the shortest path connecting them [11], [17], [18]. However, in many real-world networks, information can travel along paths that are not necessarily the shortest. Based on this idea, Estrada and Hatano (2008) first introduced the concept of communicability in the analysis of binary complex networks. This notion is a more general measure of connectivity which aims at quantifying the ease of communication between two nodes taking into consideration also non-direct physical connections. This concept has been extended to the weighted case by Crofts and Higham [19] and additionally the related notions of average path distance and of communicability centrality were defined [20], [21]. Recently, similar metrics were considered by Goni et al. [22] to quantify the density of possible detours of the shortest path. In their study, these metrics were shown to improve the power of anatomical networks to predict functional connectivity. The authors interpreted this result as an indication that signal transmission in brain dynamics does not only flow through the shortest path and this interpretation increases the interest in accounting for the contribution of indirect connections.

In the human brain network, evidence suggests that mechanisms of brain plasticity, that include the strengthening of specific connections or the recruitment of parallel and indirect connections, play an important role in learning demanding tasks or in the compensatory and reorganizational mechanisms seen after brain damage ([23]–[26]). Therefore, the concept of communicability may be useful to better understand brain plasticity and more specifically, the mechanisms of reorganization in the presence of lesions. The concept of weighted communicability was first applied to structural brain networks in the works of Crofts and colleagues [19], [27]. In these studies, communicability was found to be sensitive to changes in structural connectivity of both hemispheres after a stroke [19], [27]. More recently, Li et al. [20] have shown that in early relapsing-remitting multiple sclerosis (RRMS) patients communicability metrics were a sensitive indicator of lesions. Despite some limitations in the atlas and angular resolution, both studies suggest that communicability metrics may be more sensitive to organizational changes in the brain due to neurological and neurodegenerative disorders than standard connectivity measures. However, to date the concept of communicability has been uniquely applied in studies on patients. It remains an open question as to whether and how the description of the communicability metrics could enhance the insight of brain network topologies in general.

Therefore, in our work we first analyzed the relationship between communicability metrics and standard connectivity and distance metrics of the brain network in 19 healthy subjects. We hypothesize an additional gain in knowledge about structural brain network topology using communicability metrics complementary to standard connectivity metrics. Particularly, parallel and multiple paths may enhance integration in the network. Therefore, we expected communicability metrics to provide more information on how each node is integrated in the network. The primary aim of our work was then to explore the sensitivity of network communicability metrics using simulated lesions. The first analysis aimed at finding the best strategy to detect nodes or subnetworks sensitive to lesions, while the second one aimed at evaluating the metric changes in the presence of damage to specific nodes. These analyses showed a benefit of communicability metrics compared to standard connectivity metrics in detecting subsets of nodes vulnerable to lesions. However, the simulated lesions are modelled using a simple node or connection deletion and do not include any mechanism of reorganization. Therefore two additional analyses are presented to provide situations that are more realistic and similar to real brain injury found in neurologic disorders. First, we simulated lesions in regions similar to the ones considered in the work of Crofts et al. [27] since the comparison could highlight effects that are specifically due to reorganizational mechanisms not present in our simulations. In addition, we analyzed a small sample of stroke patients and controls with a larger variability of lesion sites and sizes as compared to the study of Crofts and colleagues. These additional analyses are not conclusive, but still add supportive evidence to our conclusions and provide interesting hypotheses on the case of real damage that should be further analyzed in larger samples.

## Methods

### 2.1 Subjects and measurements

#### 2.1.1. Ethics statements

All participants gave their written informed consent and the study was approved by the ethics committee of the Canton of Bern, Switzerland.

#### 2.1.2 Simulated lesions

Nineteen healthy young subjects participated in the study (10 women/9 men; 26.1±2.7 years). Images were acquired on a Siemens Trio 3T scanner (Siemens Erlangen Germany). The protocol for DWI used a spin echo (SE-) echo-planar imaging (EPI) with two 180° radio frequency (RF) pulses (repetition time (TR)/echo time (TE) = 6800/93 ms, matrix size = 128×128, field of view (FOV) = 256×256 mm^2^, 50 slices, slice thickness = 2 mm, gap thickness = 0 mm, pixel bandwidth 1346 Hz/pixel). Diffusion sensitizing gradients were applied at a maximal b-value of 1300 s/mm^2^ and along 42 non-collinear directions. An additional four images were acquired with b-value = 0 s/mm^2^. Each subject underwent two consecutive DWI sessions.

In addition, T1-weighted anatomical images were acquired with a 3D Modified Driven Equilibrium Fourier Transform (MDEFT) sequence [28] with a 12-channel head coil (TR/TE = 7.92/2.48 ms, matrix size = 256×256, FOV = 256×256 mm^2^, 176 sagittal slices, slice thickness = 1.0 mm, Flip angle = 16°, inversion with symmetric timing (inversion time = 910 ms), fat saturation).

#### 2.1.3 Stroke and controls

A smaller dataset of 4 stroke patients (59.8±9.5 years) and 5 controls (60.6±11.1 years) was used to determine if results on clinical data are in line with the results found with our simulations. Demographic characteristics of the four patients are given in Table 1. Subjects were measured using the same scanner and with the same sequence parameters as described above except for the resolution of DWI images.

**Table 1:** Demographic characteristics of the stroke patients included in our additional analysis.

Patient ID	Age	Stroke side	Lesion volume (number of voxels)	Lesion location	Time after stroke (d)
L1	60	Left	17	m1	83
L2	78	Left	705	m1	92
R1	53	Right	20708	m1/s1/s2/ppc	88
R2	49	Right	7044	s1/ppc	81

Location of stroke: m1 - primary motor cortex, s1 - primary somatosensory cortex, s2 - secondary, somatosensory cortex, ppc - posterior parietal cortex. The lesion volume was computed based on masks created by a radiologist.

### 2.2 Data processing and network construction

Motion and eddy current correction of diffusion weighted images (DWI) was performed using the Functional Magnetic Resonance Imaging of the Brain FMRIB software library version 4.1 (FSL, [http://www.fmrib.ox.ac.uk/fsl], Smith et al. [29]). The automated parcellation of T1-weighted images was performed in FreeSurfer (Athinoula A. Martinos Center for Biomedical Imaging, Harvard-MIT, Boston [http://surfer.nmr.mgh.harvard.edu]). Subsequently, T1-weighted images were co-registered to the first b0 and the T1-b0 transformation was also applied to atlas image using nearest neighbor interpolation.

A detailed description of the network construction can be found in the S1 Text; however to summarize, the Destrieux atlas (154 regions) was used for the analysis of simulated lesions, while for the analysis of stroke patients the Desikan atlas (86 regions) was used [30], [31]. The lower resolution was selected to increase statistical power and reduce the effects of noise in the small patient set. Labels and names of the ROIs can be found in S1 and S2 Tables. The cortical and subcortical structures defined were then used as ROIs for probabilistic fiber tracking, which was performed in FSL according to Behrens et al. [6]. The edges of the networks were defined using the connectivity indices between the two regions. In the first analysis an additional correction using the seed and target node sizes was applied. In the average network an edge between node i and node j was set, if the connection existed in at least T_avg_ = 75% of the subjects and it was weighted by the average weight over the individual networks [32].

### 2.3 Communicability and related metrics

#### 2.3.1 Communicability

When considering a network with binary adjacency matrix A, the (i,j)-th entry of the k-th power matrix A^k^ represents the number of paths of length k joining i and j. Estrada and Hatano [17] defined the concept of communicability using this property and down-weighting the contribution of longer paths. The binary communicability (Cm) between nodes i and j is given by:
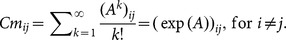



Further on, Crofts and Higham (2009) generalized the concept and obtained the weighted communicability (Cm^w^) using the weighted adjacency matrix W:
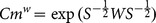
where 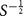 is the diagonal matrix with elements 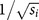 and 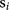 is the strength of node i. The multiplication by matrix S is a normalization step introduced to regulate undue influence of nodes with high strength. The effect of this normalization was investigated in the first analyses, while for the analyses of simulated lesions the normalized communicability was used in both the weighted and binary cases, because it was found to be more robust. Indeed, without normalization the local binary communicability has a very large standard deviation due to the strong dependency on network density. Another approach to correct this could be to threshold the networks to ensure the same density.

#### 2.3.2 Communicability Centrality

The communicability centrality (CBC) was defined in Estrada et al. [21] and measures the reduction in the global communicability of the network if a specific node is removed. Denote Cm(r) the communicability of the network without the node r, then the CBC of node r can be defined as:


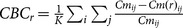
, with i≠j, i≠r,j≠r and K a normalization constant equal to the number of elements in the sum. By applying this normalization the binary CBC values lie within 0 and 1. The same definition can be used for weighted communicability.

### 2.4 Other network metrics and comparison

Communicability related network metrics were analyzed in relationship to more common metrics used in literature: strength (S^w^), distance functions and characteristic path length, betweenness (BC) and degree (Deg) centralities. In addition, the global efficiency of the network was used to evaluate the effect of lesions on the whole network. Details on the definitions of these metrics can be found in the S2 Text as well as in specific literature [10].

### 2.5 Simulated lesion analysis

Lesions of the structural connectivity network were simulated by sequentially removing nodes (and the related connections) or single edges from the network. The site of each lesion was selected randomly (*random attack*) or by using specific criteria (*targeted attack*) depending on the aim of the analysis. As many different lesion methods are presented, a summary is given in Table 2.

**Table 2:** Summary description of the different lesion methods used and their characteristics.

Aim:	Target of attacks	Selection of targets	Strategies tested
**evaluate strategies to select lesion site (see 2.5.1)**	Nodes	Single choice: *Metrics are recomputed after each removal.*	(binary and weighted)
		Hubs choice: *Removal order is based on the whole network*	Max Deg/S^w^, Max BC, Max Cm, Max CBC

**Aim:**	**Target of attacks**	**Selection of targets**	**Type of attacks**
**evaluate local metrics sensitivity to lesions (see 2.5.2)**	Nodes	Hubs	Binary: *removal of a percentage R of the connections selected randomly*
		Standard Random: *same nodes for each subject*	Weighted: *reduction of ratio R for each connection*
		Individual random: *different nodes for each subject*	
	Edges	Standard random	Binary
		Individual random	

#### 2.5.1 Targeted attacks to nodes

One aim of the analysis of the simulated structural lesions was to determine if any of the considered scalar metrics was more adequate in identifying nodes sensitive to lesions. To this end, different strategies for targeted attacks were compared and the performance of each strategy was evaluated by comparing the global network efficiency (Eff, Eff^w^) after each removal [33]. The strategies considered for the selection of the nodes to be removed were maximal Deg, Cm, BC and CBC (binary and weighted). In addition, two different target selection methods were considered. The first method was denoted as *single-choice method* and included the recomputation of the network metrics after each removal [34]. In the second method, denoted *hubs method*, the order of removal was defined once, at the beginning, based on the metric distribution of the entire network (Table 3).

**Table 3:** Summary description of Single-choice method and Hubs method for the selection of the N nodes to remove (see 2.5.1).

Single-choice method	Hubs method
For k = 1:N	Compute network metrics
- compute network metrics	Define the order O by the criteria (i.e max Deg, max Cm, max BC, max CBC)
- select the node nk to remove by thecriteria (i.e max Deg, max Cm, max BC, max CBC)	For k = 1:N
- Delete node nk	- Delete node nk = O(k)
- Evaluate Eff	- Evaluate Eff

#### 2.5.2 Small perturbations

Another aim of the lesion analysis was to understand how the different network metrics were affected by smaller perturbations of the network organization. Accordingly, lesions in which the nodes were not completely removed from the network were simulated. In particular, two different types of lesions were considered: lesions to specific nodes and lesions to single connections in the network (Table 2).

In the case of binary lesions to nodes, N = 10 nodes were selected successively and a percentage R in the range of 20%–80% of their connections was randomly selected for each subject and deleted. In weighted node lesions, all the edges of a specific node were affected by a reduction of their weight of R = 20%–80%. For every simulation, R was fixed and equal for each subject. The N nodes selected were either hubs of the right hemisphere (RH) or just N randomly selected nodes of the RH. Hubs were defined as nodes with a degree of at least one standard deviation over the mean node degree and the number N of nodes to delete was set by taking the median number of hubs of the RH over the subjects [32], [35]. For random selection, the algorithm was repeated 25 times to increase the independence of the results from the specific lesion patterns. The selected nodes were the same for each of the subjects, but the affected connections were then selected randomly. Additionally, an individual random selection method was tested, where attack sites were selected independently for each subject. Finally, in the case of lesions to network connections, at each step one single edge was removed from the network (*single edge attack*). In total Ne = 250 edges were chosen randomly. The number Ne was selected to allow a similar number of connections to be removed as compared to attacks to nodes. In the standard random selection method, the same connections were deleted in each of the subjects, while for the individual random selection method, different edges were removed. The procedure was repeated 25 times to increase independence of the results from the specific lesion pattern.

The rationale behind this analysis was to simulate the situation of a longitudinal study where measurements are taken at two time points to evaluate disease progression. Each subject underwent two consecutive diffusion imaging sequences. For each of the subjects the network, computed from one of the acquisitions (chosen randomly), was used as the first time point (baseline), while the network, obtained with the other diffusion sequence, was used in the lesion simulations to obtain the second time point. Changes in the metric distributions due to the lesions were analyzed locally comparing these two networks. The use of two different measurements makes the presented analysis more comparable to a longitudinal study in a diseased population as it introduces realistic measurement noise. The comparison of the networks before the lesion simulations is reported in the S3 Text and demonstrates that the two sets of networks were not significantly different before the simulated lesions. In a longitudinal analysis two aims may be of interest: understanding how the whole network is affected by the local lesions and the ability to detect changes as early as possible. Therefore, the sensitivity of the metrics was quantified by the total average number of significant changes from baseline and by the earlier significant change, i.e. the significant change appearing with a smaller number of lesions. The total number of changes indicates metrics that are sensitive to changes that occur in regions not directly affected by the lesions. In addition, to understand if changes have a specific pattern related to the lesion sites. In addition, local changes were analyzed with respect to the distance from the lesion. A relationship to distance may be useful in the future to interpret changes in a local metric or to map the focus of the lesion.

### 2.6 Additional analyses

#### 2.6.1 Simulated stroke lesions

Using the same method as above, weighted and binary lesions to regions around the basal ganglia were simulated in order to compare the results with the analysis in Crofts et al. [27]. Based on their results, lesions were applied to the left thalamus and caudate nodes. The lesion rate was selected randomly for each subject, in order to increase variability. The algorithm was repeated 10 times and results were averaged. As in the previous analysis, metrics of the damaged networks were compared to baseline. The analysis included the comparison of global, hemispheric and local metrics.

#### 2.6.2 Analysis of stroke patients compared to healthy controls

Global and hemispheric metrics of the stroke patients were compared to healthy controls in a qualitative analysis. In addition, the linear relationship between the difference from the control group and the distance from lesion was tested separately for every patient.

### 2.7 Software description and statistics

Graph metrics were computed using the MorphoConnect toolbox [36] and subroutines of the Brain Connectivity toolbox (https://sites.google.com/site/bctnet/). For visualization of the lesions in the brain networks BrainNet Viewer was used (http://www.nitrc.org/projects/bnv/, Xia et al. [37]). In order, to compare different strategies for target selection (Section 2.5.1), the efficiency decay curves were compared using a permutation test. In particular, the set of curves for two selection criteria were separated randomly into two groups and the sum of differences in efficiency over N lesions was used as a statistic. In total, 5000 permutations were performed. In addition, the global efficiency distributions were tested for differences using paired t-tests after a given number of attacks. Also, paired t-tests were used for global and local network metrics. For the analysis of local metrics False Discovery Rate (FDR) correction was applied for multiple testing [23]. Additionally, correlations were computed to analyse the relationship between the standard connectivity, distance and communicability matrices as well as for the relationship between local changes and distance from lesion. For continuous variables, the Pearson's correlation coefficient was used, while in the presence of ordinal variables or when the relationship was not linear, the Spearman's coefficient was preferred. For all analyses, the corrected significance threshold was set at p<0.05.

## Results

### 3.1 Communicability in the healthy brain structural network

In the first step of our analysis, we assessed the relationships between communicability, standard connectivity and distance function in order to understand how communicability is related to more commonly used measures. These relationships were analysed for the average network of all subjects as well as for individual networks and results were consistent.

#### 3.1.1 Relationship between standard connectivity and communicability

**Figure 1:**
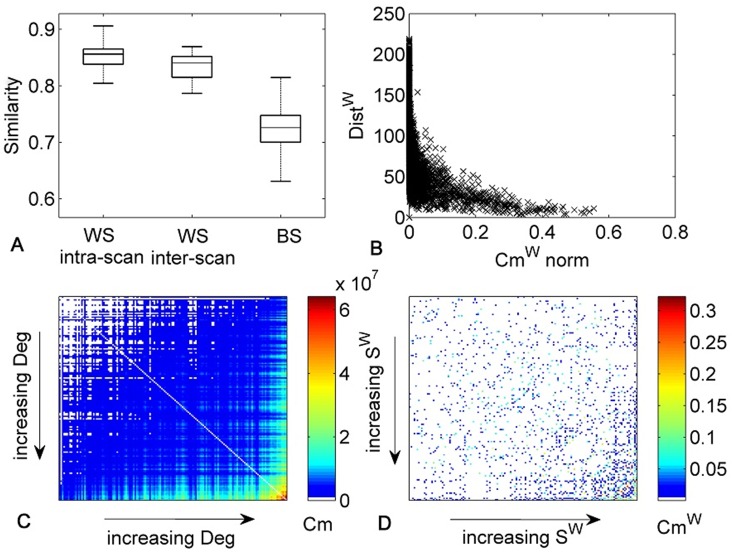
Relationship between communicability, standard connectivity and distance measures. A) Boxplot of the correlations of C and Cm^w^ respectively within subjects (WS) intra-scan, WS inter-scan, and between subjects (BS). B) Scatter plot of Cm^w^ and Dist^W^ matrices for the average network. C) Binary communicability assortativity matrix, i.e. Cm distribution among nodes with increasing Deg. D) Weighted communicability assortativity matrix, i.e. Cm^w^ distribution between nodes with increasing S^w^.

The correlation between standard connectivity (C^w^) and normalized communicability Cm^w^ was very high for the existing connections. In particular, in the average network, the Pearson's correlation coefficient was of r = 0.82 and on average over the subjects r = 0.83±0.03 (Fig. 1A). Both C^w^ and Cm^w^ also show high correlations between subjects (r_C_ = 0.74±0.06, r_Cm_ = 0.82±0.03).

#### 3.1.2 Distribution of communicability over nodes

**Figure 2:**
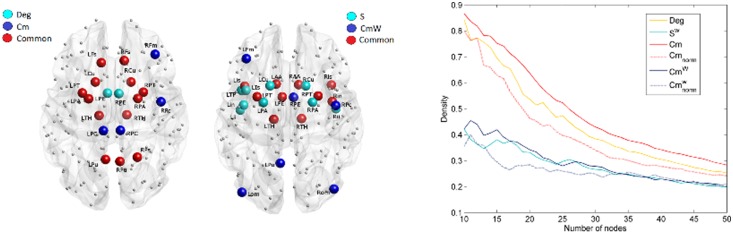
Analysis of hubs and nodes with highest communicability. A) maps of the nodes with highest Deg and/or Cm. B) maps of nodes with highest S^w^ and/or Cm^w^ (normalized). The metric values for the nodes represented are at least one standard deviation (SD) over the average value. C) Density variation among the nodes with highest Deg, Cm, S^w^, Cm^w^.

Compared to the other metrics considered, normalized communicability was more equally distributed over all nodes. Indeed, over the whole average network the standard deviations of the L^2^-normalized metrics were respectively 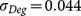, 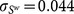, 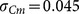, 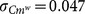, 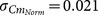, 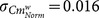. This effect indicates that the normalization diminishes the influence of hubs in the communicability as described in Crofts and Higham [19]. In Fig. 1C and 1D, the distribution of communicability among nodes ordered with increasing Deg or S^w^ is shown. For binary Cm, higher Cm was found between nodes with higher Deg. In the weighted case, this relationship was less evident, but higher communicability was more frequent among nodes with higher S^w^. Also after normalization higher communicability was found between the 50 nodes with highest degree as compared to the communicability among the 50 nodes with lowest degree or between nodes with highest degree and nodes with lowest degree. This relationship between Deg/S^w^ and communicability is denoted as positive assortative communicability [17]. Nonetheless, Fig. 2 shows that in the maps of the nodes with highest Deg, S, Cm and Cm^w^ some differences are present. Detailed results for each metric are reported in S3 Table and S1 Fig. In addition, the density of connections among the nodes with higher Deg, S^w^, Cm, Cm^w^ was compared and the highest density was found with Cm, when no normalization was applied (Fig. 2).

#### 3.1.3 Relationship between distance function and communicability

A negative correlation was found between Dist^w^ and normalized Cm^w^. Specifically, in the average network, the Spearman's correlation coefficient was of r = –0.72 (Fig. 1B) and on average over the subjects r = –0.69±0.02. However, the relationship between Dist^w^ and Cm^w^ was not linear (Fig. 1B). The correlation coefficient between Dist^w^ and C^w^ is r = –0.42.

### 3.2 Analysis of simulated lesions

#### 3.2.1 Targeted attacks to nodes

**Figure 3:**
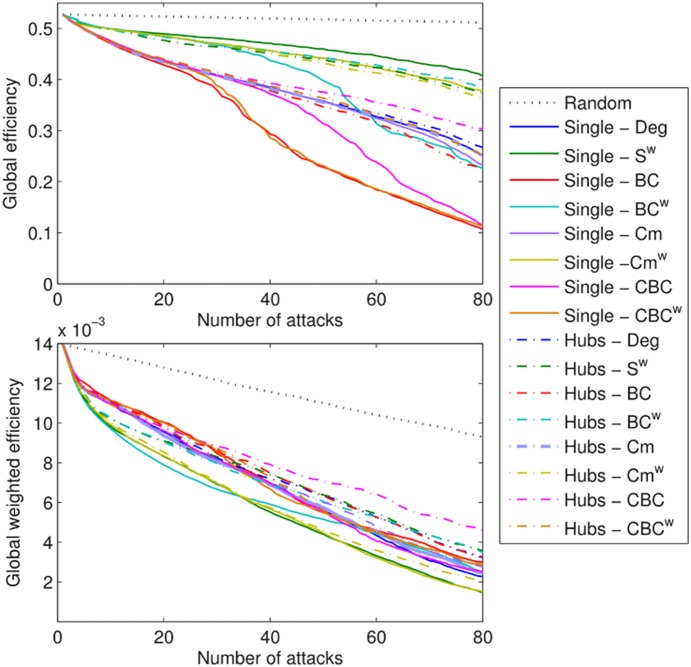
Average efficiency decay (Eff, Eff^w^) curves over subjects for the different target selection strategies. Curves are reported for a total of N = 80 consecutives attacks.

In total, up to 80 nodes were removed from each of the individual networks. The average efficiency (Eff, Eff^w^) for each of the strategies is shown in Fig. 3. Results show that overall the single-choice methods had a stronger effect on the global efficiency of the network (Fig. 3, Perm test: p<0.0001). When Eff was considered, BC-Single and CBC^w^-Single were the most effective strategies for the identification of nodes that are responsible for the network efficiency. When more than 15 nodes were removed, the strategy CBC-Single was significantly worse than BC-Single and CBC^w^-Single (t-test: p<0.0001, Perm test: p<0.001), however it was also significantly better than all the other strategies (Perm test: p<0.003 VS Hubs-BC). When Eff^w^ was considered the effects were less evident; however, BC^w^-Single, S^w^-Single and Cm^w^-Single were significantly more effective than the other strategies for target selection (Fig. 3). Remarkably, Cm^w^-Hubs was similarly effective. In particular, the efficiency obtained by Cm^w^-Hubs was not significantly different than with S^w^-Single (Perm test: p<0.21, n.s.), but it was from all other Hubs strategies (Perm test: p<0.02 CBC-Single).

#### 3.2.2 Small perturbations- Sensitivity of the local metrics

The aim of the analysis of smaller perturbations was to understand how local metrics are affected by lesions and if some of the metrics are more sensitive.

**Figure 4:**
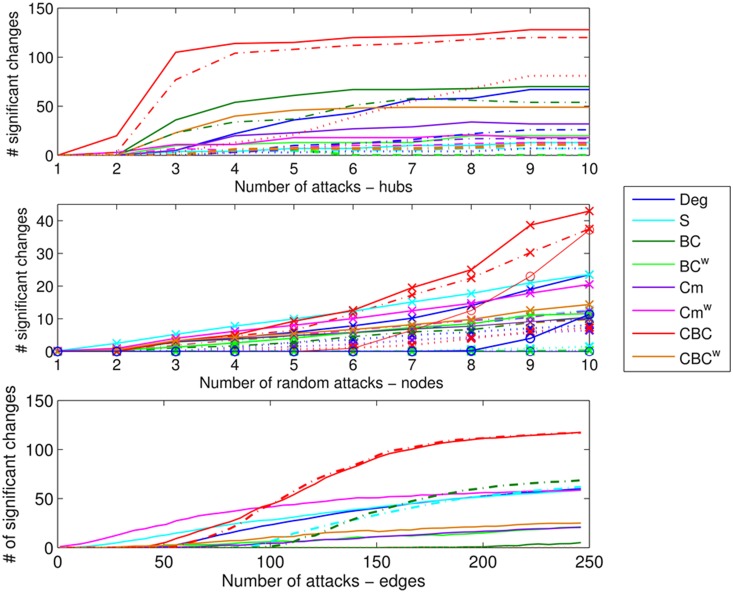
Local changes due to simulated lesions. Top: number of significant local changes for the various metrics when hubs were targeted for binary lesions. The line types indicate the rate of deleted connections: solid lines R = 0.8, dashed lines R = 0.5 and dotted lines R = 0.2. Center: average number of significant local changes over 25 repetitions for the various metrics when random nodes were targeted for binary lesions. The line types indicate the site selection and rate: solid lines with cross markers for same nodes for all subjects and rate R = 0.8, dashed lines with cross markers for same nodes for all subjects and R = 0.5, dotted lines for with cross markers for same nodes for all subjects and R = 0.2 and solid lines with circle markers different nodes for each subject R = 0.8. Bottom: average number of significant local changes over 25 repetitions for the various metrics when edges were targeted for binary lesions. The line types indicate if the same edges (solid lines) or different edges (dashed lines) were selected for each subject.

Over all types of binary lesions, CBC was the most sensitive metric (Fig. 4 and S4 Text) in terms of number of significant changes. However, note that binary metrics will not be affected in the case of weighted lesions (S2 Fig.). With the exception of hub lesions, the earlier changes were seen for weighted metrics such as S^w^ and Cm^w^. Specifically, when single edges were affected, Cm^w^ was the only metric already showing significant changes when only 3 edges had been removed (Fig. 4). However, when different edges were removed randomly for each subject, only Deg, S^w^ and CBC showed significant changes, the latter showing the earlier and greater changes.

#### 3.2.3 Small perturbations- Location of changes

**Figure 5:**
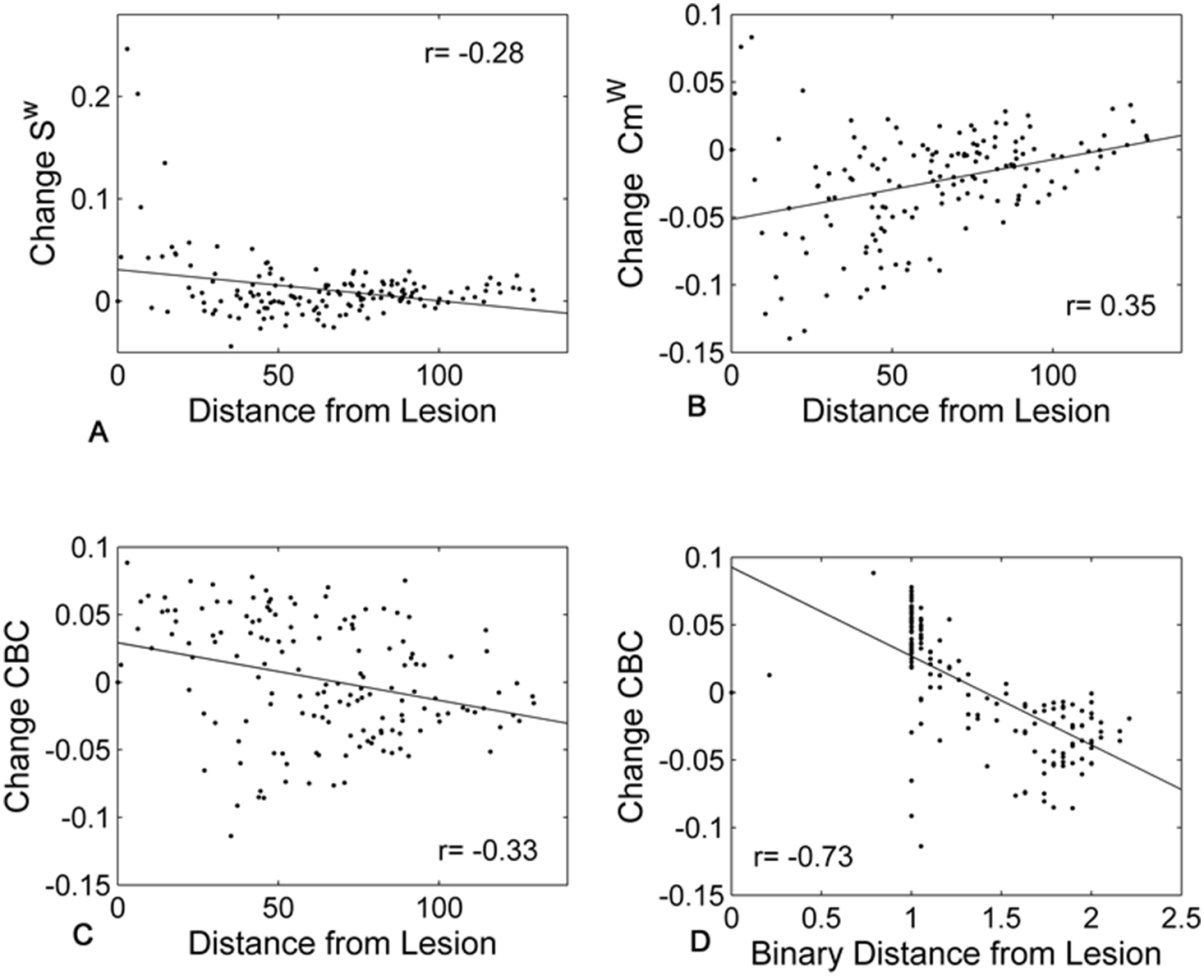
Relationship between the average local changes over subjects and the distance from the lesion site (after 5 attacks to hubs nodes). Each dot in the figure represents a node in the average network. A positive change indicates a reduction in the metrics after the lesions. Scatter plots are reported for S^w^ (A), Cm^w^ (B) and CBC (C) against weighted distance and for CBC against binary distance (D).

In order to understand whether some metrics are more prone to show changes remote to the lesion site, the local changes were analyzed in relation to the distance from the lesion focus. This analysis provides additional information on the relationship between the changes and the pattern of lesions applied. The situation with 5 lesions to hubs was considered and, in general, correlations strengthened when a larger number of attacks was applied. When the average change and distance were considered, several metrics showed relatively strong correlations (Table 4). CBC was the metric showing the highest individual coefficients. A negative correlation with an increase in CBC in the contralesional hemisphere was found and the average Spearman’s correlation coefficient with the binary distance over all subjects was of r = -0.42 after 5 attacks (Fig. 5, C and D, significant for all subjects).

**Table 4:** Correlation coefficients between local metric changes and (weighted and binary) distance from lesions are reported.

Number of attacks		1		3		5	
Correlation		Individual	Average	Individual	Average	Individual	Average
**Deg**	DistW	0.03±0.14	0.12	−0.04±0.15	−0.11	−0.09±0.15	−0.21
	DistB	−0.07±0.14	−0.32	−0.18±0.14	−0.55	−0.22±0.13	−0.60
**S^w^**	DistW	0.01±0.20	−0.03	−0.11±0.18	−0.39	−0.10±0.18	−0.29
	DistB	−0.06±0.15	−0.28	−0.11±0.13	−0.40	−0.11±0.13	−0.43
**BC**	DistW	0.11±0.09	0.23	0.20±0.08	0.34	0.18±0.10	0.39
	DistB	0.12±0.10	0.38	0.27±0.09	0.69	0.29±0.07	0.71
**BC^w^**	DistW	0.11±0.09	0.23	0.06±0.11	0.11	0.07±0.11	0.17
	DistB	0.04±0.10	0.14	0.12±0.07	0.30	0.11±0.06	0.23
**Cm**	DistW	0.09±0.09	0.36	0.15±0.10	0.49	0.16±0.10	0.50
	DistB	0.06±0.07	0.18	0.13±0.06	0.53	0.15±0.05	0.60
**Cm^w^**	DistW	0.07±0.06	0.14	0.17±0.07	0.35	0.18±0.06	0.35
	DistB	0.06±0.08	0.21	0.17±0.08	0.55	0.14±0.08	0.47
**CBC**	DistW	−0.03±0.22	−0.08	−0.18±0.18	−0.32	−0.23±0.16	−0.33
	DistB	−0.18±0.19	−0.60	−0.40±0.13	−0.74	−0.42±0.08	−0.73
**CBC^w^**	DistW	0.05±0.10	0.31	0.20±0.09	0.33	0.20±0.10	0.37
	DistB	0.08±0.07	0.21	0.22±0.08	0.63	0.23±0.07	0.64

For binary distance the Spearman’s correlation coefficient is reported (rows DistB), while for weighted distance the Pearson’s correlation coefficient is given (rows DistW). Mean and standard deviations of individual correlations are reported (Individual) as well as the correlation coefficients between the average change and average distance from lesions (Average).

This negative relationship was confirmed in the case of random lesions (S5 Text), although not as strongly. Note that lesion sites were excluded from both the computation of the correlation coefficients and the scatter plots reported in Fig. 5.

The location of the most significant changes (excluding the damaged nodes) were analyzed in order to understand if there were nodes more strongly affected by network lesions in general. Overall, orbital and frontal medial cortical areas showed the strongest changes in more than one third of the cases and the accumbens area, precentral and occipital superior cortex in over one fourth. Moreover, the orbital cortex showed the most significant changes especially when communicability metrics were considered. The details of this analysis are reported in the S5 Text.

### 3.3 Additional analyses

The two additional analyses used to approach more realistic situations of brain injury are presented in this section. Specifically, the results of the simulated lesions near the basal ganglia are given in Section 3.3.1 for comparison with the analysis in Crofts et al. [27], while in Section 3.3.2 the results of the stroke patients dataset are reported.

#### 3.3.1 Analysis of simulated stroke lesion

The global metric differences were significant for Deg (p<0.05) and CBC (p<0.02) with both showing a reduction after lesion. Additionally, in the ipsilesional hemisphere a significant reduction was found for Cm^w^ (p<0.01) and CBC (p<0.01) while in the contralateral hemisphere a significant increase for BC (p<0.04), BC^w^ (p<0.03), Cm (p<0.01) and CBC (p<0.02) was detected.

The analysis of local changes showed that Cm^w^ was the most sensitive metric with 8 nodes showing significant differences. However, CBC showed more variability over the repetitions, indicating that it may be the most sensitive metric to the exact pattern of damaged connections. Significant changes in Cm^w^ were found in the contralateral frontal superior region and also in the hippocampus, fronto-orbital, postcentral and rectus gyrus of the ipsilateral hemisphere. Among the other metrics, significant changes were often found in the frontal-orbital cortex of both hemispheres, in regions near the basal ganglia (putamen, nucleus accumbens, hippocampus, amygdala) and in the frontal regions. Also, in the contralesional hemisphere regions of the insular and cingulate cortex changes were found. In general, the more significant changes were found in the lesioned hemisphere. Details on the significant regions for each metric are given in S6 Text.

#### 3.3.2 Analysis of stroke patients versus healthy controls

**Figure 6:**
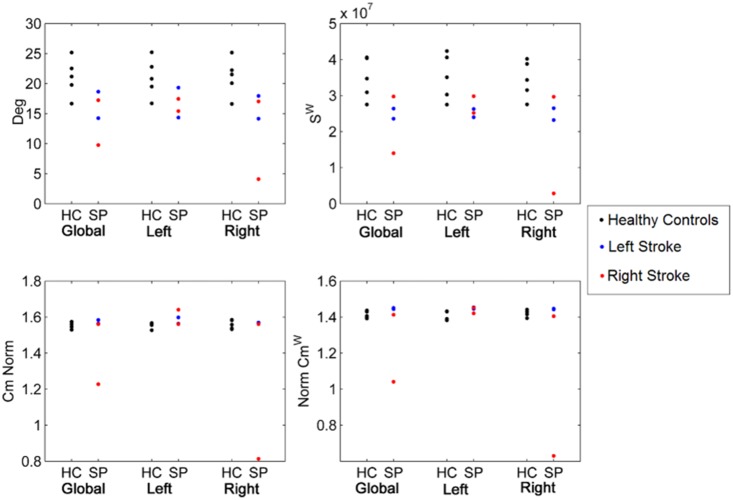
Global and hemispheric network metrics of Deg, Cm, S^w^ and Cm^w^ for healthy controls (HC) against stroke patients (SP).

Fig. 6 shows a reduction for all patients in Deg and S^w^ in both hemispheres independently on the lesion site. Both Cm and Cmw show a slight increase in all patients except one that shows a large reduction on the ipsilesional hemisphere. Plots of the other metrics (BC, BC^w^, CBC, CBC^w^) are reported in the S6 Text.

**Figure 7:**
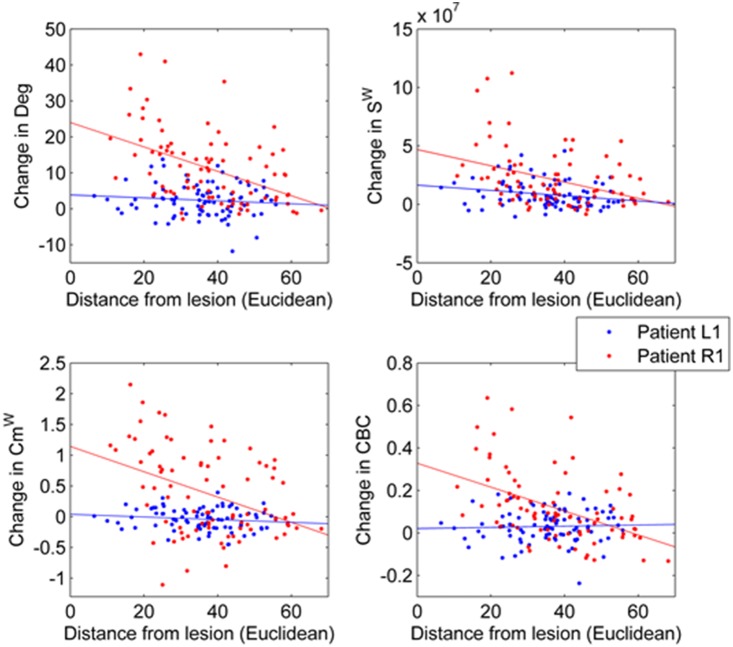
Local correlations between changes ((A) Deg, (B) S^w^ (C) Cm^w^ (D) CBC) from the control group and distance from lesion for stroke patient L1 (largest lesion) and R1 (smallest lesion). Crosses indicate local values for patient L1, while dots are associated to local values of patient R1. Lines indicate the least square lines associated to each relation.

Table 5 shows the correlation coefficients between the Euclidean distance from lesion and the difference from baseline (healthy control group). Correlations for degree and strength were stronger than in the case of the simulated lesions, probably because, in this case, the lesion was not excluded from the analysis. S^w^ shows the most consistent correlation for all patients, while CBC, Cm^w^ and Deg seem to show stronger correlations in the patients with larger lesions (Fig. 7).

**Table 5:** Correlation coefficients of local differences from the control group with the Euclidean distance from the lesion are reported for each stroke patient and each network metric.

Patient ID		L1	L2	R1	R2
**Deg**	DistE	−0.11	−0.13	−0.46	−0.46
**S^w^**	DistE	−0.22	−0.22	−0.39	−0.25
**BC**	DistE	−0.05	0.09	−0.11	0.10
**BC^w^**	DistE	0.08	−0.02	−0.16	0.04
**Cm**	DistE	−0.11	−0.04	−0.43	−0.08
**Cm^w^**	DistE	−0.14	−0.08	−0.41	−0.24
**CBC**	DistE	0.04	0.06	−0.47	−0.31
**CBC^w^**	DistE	−0.04	0.11	−0.14	0.04

## Discussion

The current study presents an explorative analysis of communicability metrics of the structural brain network organization in healthy controls. Driven by previous results suggesting the sensitivity of communicability in the case of lesions, our work had two major aims. Firstly, the descriptive analysis of communicability metrics in the healthy brain was conducted. Secondly, the study aimed at understanding the changes and sensitivity of communicability metrics compared to standard network metrics in the presence of simulated focal lesions. In particular, to our knowledge communicability centrality was applied to brain structural networks for the first time [21]. The results on these two major topics are discussed in this section and additionally, results on stroke patients are used to qualitatively explore the relationship between simulated network lesions and real brain injury.

### 4.1 Communicability metrics in the healthy structural brain

The concept of communicability was first introduced by Estrada and Hatano [17] and is based on the idea of considering all possible paths in order to quantify the communication flow in a network. This idea is interesting, because even if we would generally expect information to flow through the most direct connections, it is also true that subnetworks in the brain may work in parallel when solving a specific task [8], [38]. Hence it is possible that more indirect paths, linking all the regions of a subnetwork, may be more efficient for a specific task. Also, Goni et al. [22] recently presented an analysis with two other communication metrics to account for the possible alternative paths around the shortest path. Their results showed better coupling of functional and structural connectivity when communication metrics were considered. This suggests that indirect structural pathways might also contribute to functional connectivity. Thus, in order to disentangle the relationship between function and structure it is important to consider the complexity of the network structure and how the shortest path is embedded in the whole network. In addition, evidence indicates that reorganization following brain injury may also affect remote regions with an increased recruitment of parallel existing pathways or “latent” connections. This suggests that indirect connections may be important also for reorganizational mechanisms [27], [39].

Despite the existence of hubs in the brain, the network is highly efficient indicating that all the nodes and paths are well integrated in the complex system. This integration appears to be highlighted by normalized communicability metrics which are more equally distributed over the nodes than standard connectivity metrics. The correlation between strength and communicability was high and the brain structural network was found to have positive assortative communicability, which means that the highest communicability is found between nodes with high degree (hubs) [17]. This property may seem to be obvious, however in Estrada et al. [21] it is shown that it is not satisfied in all networks especially when high clustering is present. These positive associations between standard connectivity and communicability suggest that the communicability matrix store the whole information about direct connections. However, two elements indicate that communicability contains useful additional information on the whole network organization. Firstly, a stronger (negative) correlation between distance and communicability was found suggesting that when taking into consideration all paths more information about the relationships between all pairs of nodes is stored. Secondly, the fact that the nodes with the highest communicability were found to form a core that was more densely connected than hubs with highest degree and that a high communicability between hubs was also detected may indicate the importance of parallel paths in the core of the network. In Goni et al. [22] the authors suggest that the higher predictive power of communication measures as compared to standard connectivity may suggest that functional dynamics of signal transmission include diffusion or spreading dynamics and that therefore noise and dispersion of signal is increased in hubs nodes. From this perspective, high communicability between hubs may be seen as a protective mechanism from errors in transmission.

In addition, from a methodological point of view, in connected networks communicability is defined for each pair of nodes allowing the analysis of this metric for the whole network, a node or a single direct or indirect connection. Communicability metrics are however more complex and also involve indirect connections making the results more difficult to interpret.

### 4.2 Analysis of simulated lesions

A large number of studies approached the topic of lesions in complex networks and in particular in the brain structural network by considering cases of real damage, simulated lesions or both [34], [40]–[44]. This topic is of interest for several reasons. Firstly, the possibility to deepen the understanding of the link between structural network damage and functional outcome as well as the mechanisms of brain plasticity that favour recovery after lesion. Secondly, this type of study enables the further understanding of the complex organization of the brain structural network as well as the role of hubs and other nodes. And finally, the integration of the analyses of real injury and simulation may help to clarify mechanisms of damage and recovery specific to neurological diseases.

In our study, simulated lesions were used with two main objectives: to evaluate the best strategy to identify nodes and subset of nodes sensitive to lesions and to explore the sensitivity of the different local metrics over the network in the presence of focal lesions. The first analysis aims at predicting the outcome of a lesion in a specific region of the brain. Indeed, the global efficiency of the network relates to its functional integration and several theoretical and clinical studies showed a relationship between network failure and efficiency loss [13], [45]–[47]. Our analysis confirms the results of Alstott et al. [34] showing that betweenness centrality metrics perform better in the detection of nodes sensitive to lesions in binary networks compared to degree metrics. However, our analysis also showed that betweenness centrality and communicability centrality performed equally well for this scope. When weighted efficiency was considered, both communicability and strength performed as effectively as betweenness centrality for the selection of one single node (Single-choice method). However, for the selection of a subset of nodes (Hubs method), weighted communicability outperformed all the other strategies. Recently, many studies have suggested a central role of hubs in the brain structural network. In particular, van den Heuvel and Sporns [32] showed that the brain structural network has a rich-club structure where hubs form a densely connected backbone and cover a large proportion of the total communication costs [48]. The existence of a core of nodes highly connected has been suggested to favour integrative information processing and efficient communication [49]. Also, due to the high density of connections, it has been suggested that hubs may act as a collective [32]. Our result suggests that weighted communicability may be even more efficient than the degree in detecting the central core of nodes that is responsible for the well-functioning of the network. Indeed, efficiency was strongly affected by removing subsets of nodes with highest communicability. This is also supported by our findings of higher density among nodes with higher communicability compared to hubs with higher degree or strength.

The aim of the second analysis was to understand how the different metrics change in the presence of focal lesions and relates, for example, to the possibility of detecting changes in the early stages of diseases. Previously, two clinical studies using communicability metrics for the analysis of brain structural networks suggested that these metrics might be more sensitive than standard connectivity metrics in detecting network changes in patients with brain injuries. In particular, Crofts et al. [27] reported changes in weighted communicability in both the ipsilesional and contralesional hemisphere of stroke patients and showed that communicability was the best metric to separate patients from controls. More recently Li et al. [20] found local changes in communicability metrics in early relapsing-remitting multiple sclerosis patients that correlated with the group lesion map. These results suggest that communicability metrics are more sensitive to lesions and reorganizational changes following injury. The analysis of binary lesions highlighted large and distributed changes in the communicability centrality as well as an interesting correlation with the distance from the lesion which showed that CBC was decreased near the lesion and increased in the opposite hemisphere. This result highlights the asymmetry that is created by lesions in one hemisphere and the increased relative importance of the opposite hemisphere. However, note that CBC is a binary metric and hence will not be sensitive to lesions that only affect the weight of a connection. The higher sensitivity of weighted metrics to smaller lesions is also seen in our analysis in the cases of random lesions to nodes and connections. In particular, comparing attacks to hubs and random attacks to nodes and connections, it appears that strength and communicability are more sensitive to smaller changes, while centrality metrics are more strongly affected when a hub is damaged. Changes in strength are restricted to nodes around the lesion while changes in communicability are more largely distributed over the ipsilateral hemisphere. Overall, significant changes in the contralesional hemisphere are seen only for critical lesions, especially those in the subcortical hubs, and in the contralateral hemisphere CBC is clearly more affected than other metrics. In summary, our analysis of simulated lesions showed that, despite the larger variability of local metrics due to noise [35], [50], these metrics are able to show significant changes that relate to the distance from the lesions, their number and importance.

### 4.3 Additional analyses and relation to real brain injury

Our study of simulated lesions enabled the analysis of the effects on efficiency and the local changes of network metrics. However, it is difficult to relate this type of analysis to real brain injury, because reorganizational mechanisms are neglected in the simulations and the full pattern of changes in the presence of neurodegenerative or neurologic disorders still remains unknown. Such mechanisms include an increased recruitment of parallel existing pathways or “latent” connections, the reorganization of distant sites as well as increased expression of sodium channels and synaptic changes [24] and therefore communicability metrics may be particularly sensitive to reorganizational changes after a lesion. In order to discuss the differences of simulated lesions and the analyses of real brain damage, simulated lesions were also used to create a series of lesion similar in site to the subcortical strokes analysed in Crofts et al. [27]. In our analysis of simulated lesions several global metrics showed differences also in the contralesional hemisphere suggesting a change in the overall network organization, however Cm^w^ significantly changed only in the ipsilateral hemisphere. Weighted communicability was the most sensitive metric, but significant local changes in communicability were mostly found in the ipsilateral hemisphere. In opposition, Crofts and colleagues found significant changes in communicability in both hemispheres of real stroke patients enabling also a separation of patients and healthy controls using only data from the contralesional hemisphere. Despite our lesion model being rather simple, this difference suggests that the reorganizational changes after a lesion that were omitted in our simulations have an important effect in the contralateral hemisphere that can be captured by weighted communicability.

In addition, a small sample of stroke patients was compared to healthy controls in order to gain insights on changes in the brain structural network of stroke patients with large differences in lesion sites and sizes. Considering the reduced number of subjects analysed, this analysis is only considered qualitatively and further studies will be needed to confirm these first results. At the hemispheric level, in all patients except one, communicability metrics show a tendency to increase despite a loss in connectivity (Deg, S^w^). This result suggest that in the case of real brain injury reorganizational changes can be captured by this network metric and that a differential behaviour might appear related to the seriousness of the lesion. Finally, individual correlations between the difference from the control group and the distance from lesion suggest that local metrics are sufficiently consistent across healthy subjects to be sensitive to changes due to local brain damage.

### 4.4 Methodological limitations

There are several methodological limitations to the current work related to both the general framework of connectome analyses and the specific procedure of simulated lesions.

In literature, criticism on the analysis of brain structural networks mostly relates to three major points: the capability of current tractography methods to reliably reconstruct the network, the difficulty of defining a meaningful weight for the connections and the dependency of the network metrics to the atlas and resolution selected [51], [52]. In our analysis, we decided to construct the networks using methods that are commonly used in literature for this type of analysis in order to be able to compare our results to the studies that have already used communicability on patients [20], [27]. Despite the drawbacks of this methodological pipeline, we assume that the tractography algorithm and the weight selected would not affect our principal conclusions on the benefits of adding communicability metrics in the anaylsis of the brain structural network. Similarly, concerning the possible effects of the atlas selected and the number and size of nodes, we consider that while the exact location of changes and the values of metrics may not be repeatable with different atlases, the overall conclusions on the sensitivity and properties of communicability metrics will not be affected by these methodological choices. An objective analysis of these issues will be addressed in a future work.

In addition, several studies analysed the reliability of the standard network metrics reporting a relatively high reliability for the global networks and higher variability in local network metrics [35], [50], [53]. In our analysis such variability was accounted for because two separate measurements were used; hence enabling to conclude that the sensitivity of the local metrics is sufficient to detect changes in the presence of lesions. Nevertheless, the presence of noise was evident considering for example the local change in strength or the comparison at baseline. A more severe threshold on probabilistic tractography could be beneficial to reduce the noise, although it could also obscure the real variability between subjects. In our analysis of healthy subjects the threshold used was relatively low in order to maintain the intra-subject variability and this may have affected some binary metrics as they are more sensitive to false positives. Using a higher threshold may make these metrics more robust. Nonetheless, CBC was found to be useful to detect local changes, suggesting that the level used was reasonable also for binary metrics.

Finally, as mentioned above, our lesion model is rather simple. Similar models have been used in literature to understand the effect of lesions in specific regions and the topology of the brain structural network [34], [42]. In our additional analyses (Section 4.3), an effort is made to discuss our results using simulated lesions relative to real injury. However, due to the limitations of the lesion model and the reduced sample size of the patient group a rather indirect approach is used in our discussion and our analyses can only provide some preliminary hypotheses that should be further assessed. In particular, more specific models of neurologic disorders are necessary to understand how the changes detected in network analysis studies relate to neurophysiological changes.

## Conclusion

This study presents the first analysis of communicability metrics in the healthy connectome. In addition, the brain structural networks were used to analyse the effect of simulated focal lesions on the distribution of local metrics and global network efficiency. Finally, two further analyses were used to discuss the differences between simulated lesions and real brain injury, considering the specific case of stroke patients. In the healthy brain higher communicability was found between nodes with high degree and local communicability correlated well with the standard connectivity. However, the communicability distribution and its correlation to network distance measures suggest that communicability also stores information on integration properties of the network. In addition nodes with highest communicability were found to be more densely connected than the ones with highest degree or strength and this could be useful to define the core of the network. Together with the results on the sensitivity of communicability metrics in the case of lesions, these results support our hypothesis that the measure of communicability may enhance the insight of brain network integration properties. The simulated lesion analysis included measurement noise, and thus allowed for the conclusion that local network metrics are sufficiently sensitive to detect changes due to focal lesions. Results showed the potential of weighted communicability to detect subsets of nodes more vulnerable to lesions and its sensitivity to a small number of random lesions to nodes and connections.

## Supporting Information

S1 Fig **Bar plots of metrics for hubs of the average network.**
(DOCX)

S2 Fig **Number of local changes for weighted lesions.**
(DOCX)

S1 Table **Labels and associated ROI names in the Destrieux atlas.**
(DOCX)

S2 Table **Labels and associated ROI names in the Desikan atlas.**
(DOCX)

S3 Table **Hubs in the average network.**
(DOCX)

S1 Text **Data processing and brain structural network construction.**
(DOCX)

S2 Text **Graph metrics definitions.**
(DOCX)

S3 Text **Group comparison at baseline.**
(DOCX)

S4 Text **Analysis of the effects of variability in 150 of random attacks to nodes.**
(DOCX)

S5 Text **Analysis of the distributions of local changes.**
(DOCX)

S6 Text **Analysis of simulated stroke lesions and stroke patients.**
(DOCX)
