# Supplementary material for: Validation of Network Communicability Metrics for the Analysis of Brain Structural Networks
Source: PLoS One. 2014 Dec 30;9(12):e115503. doi: 10.1371/journal.pone.0115503 (PMC4280193; doi:10.1371/journal.pone.0115503)
Supplement: S1 Fig — Bar plots of metrics for hubs of the average network. (DOCX) [file pone.0115503.s001.docx]

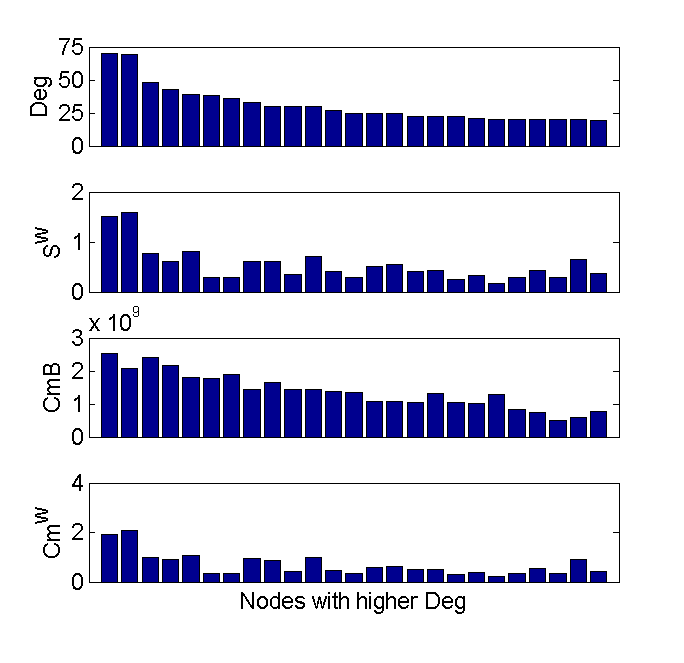


Figure S1: bar plot of metrics of the average network for the 25 nodes with higher Deg. The nodes with highest Deg are: Putamen (R/L), Thalamus (R), Pallidum (R), Thalamus (L), Frontal sup (R/L), Pallidum (L), Caudate (R), Precuneus (R), Caudate (L), Precuneus (L), Parietal sup (R), Pericallosal (R/L), Front inf Opercular (R), Precentral (R), Parietal sup (L), Parietal inf Supramar (R), Paracentral (R), Occipital sup (R), Orbital gyrus (L), Parietal inf Supramar (L), Circ insular sup (L), Occipital inf (R).
