# Supplementary material for: Validation of Network Communicability Metrics for the Analysis of Brain Structural Networks
Source: PLoS One. 2014 Dec 30;9(12):e115503. doi: 10.1371/journal.pone.0115503 (PMC4280193; doi:10.1371/journal.pone.0115503)
Supplement: S2 Fig — Number of local changes for weighted lesions. (DOCX) [file pone.0115503.s002.docx]

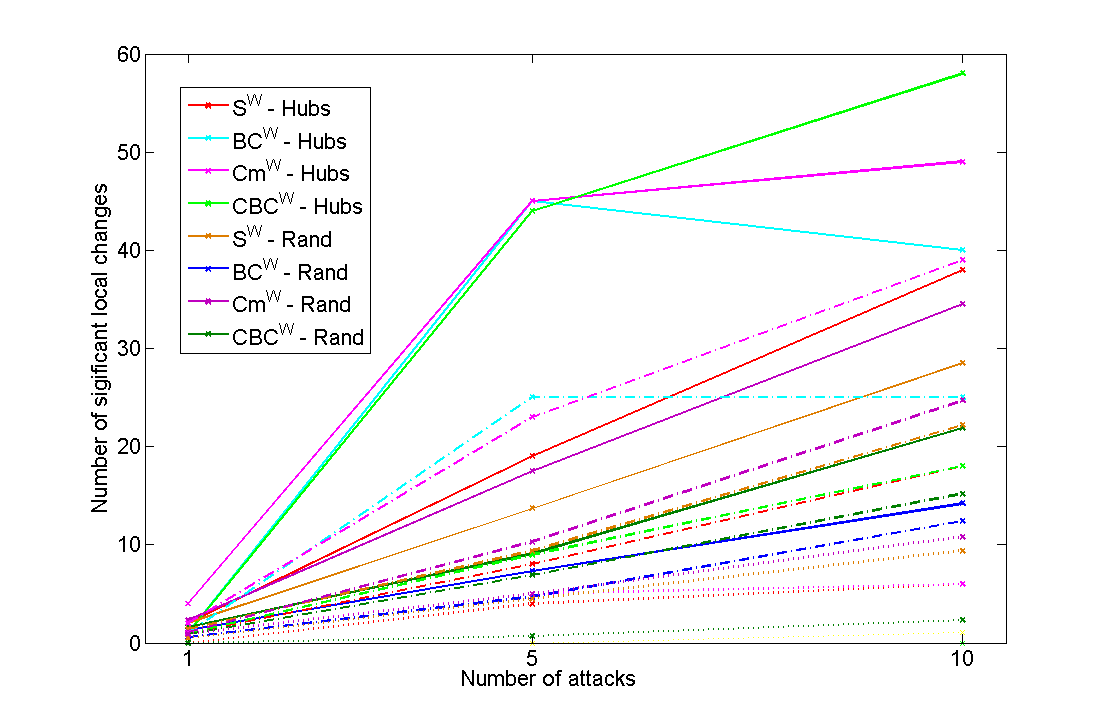


Figure S2: number of significant local changes (FDR corrected) with increasing number of weighted lesions to hubs or random nodes. Solid lines are for R=0.2, dash-dotted lines for R=0.5 and dotted lines for R=0.8. The average number of significant changes in local weighted metrics after weighted lesions to specific sites is reported. Overall, Cm^W^ is the most sensitive metric. Indeed, centrality metrics (CBC^W^ and BC^W^) are more sensitive in the case of attacks to hubs nodes, but they are less affected in the case of attacks to random nodes. No significant change was found for the binary metrics. Overall, for weighted metrics, the results of the analysis of weighted lesions are in line with the case of binary lesions, while binary metrics are not affected by weighted lesions.
