# Supplementary material for: Validation of Network Communicability Metrics for the Analysis of Brain Structural Networks
Source: PLoS One. 2014 Dec 30;9(12):e115503. doi: 10.1371/journal.pone.0115503 (PMC4280193; doi:10.1371/journal.pone.0115503)
Supplement: S1 Table — Labels and associated ROI names in the Destrieux atlas. (DOCX) [file pone.0115503.s003.docx]

| **ROI label** | **ROI Name** | **ROI label** | **ROI Name** |
| --- | --- | --- | --- |
| TH | Thalamus-Proper | Tl | ctx_ G_temp_sup-Lateral |
| Cu | Caudate | TP | ctx_ G_temp_sup-Plan_polar |
| PT | Putamen | Tt | ctx_ G_temp_sup-Plan_tempo |
| PA | Pallidum | Tm | ctx_ G_temporal_middle |
| HP | Hippocampus | Lh | ctx_ Lat_Fis-ant-Horizont |
| AY | Amygdala | Lv | ctx _Lat_Fis-ant-Vertical |
| AA | Accumbens-area | Lp | ctx_ Lat_Fis-post |
| FT | ctx_ G_and_S_frontomargin | PO | ctx_ Pole_occipital |
| oi | ctx_ G_and_S_occipital_inf | CL | ctx_ S_calcarine |
| PC | ctx_G_and_S_paracentral | CE | ctx_ S_central |
| SC | ctx_ G_and_S_subcentral | CM | ctx_ S_cingul-Marginalis |
| tF | ctx_ G_and_S_transv_frontopol | ia | ctx_ S_circular_insula_ant |
| CA | ctx_ G_and_S_cingul-Ant | ii | ctx_ S_circular_insula_inf |
| Ca | ctx_ G_and_S_cingul-Mid-Ant | Is | ctx_ S_circular_insula_sup |
| Cp | ctx_ G_and_S_cingul-Mid-Post | tv | ctx_ S_collat_transv_post |
| Cd | ctx_ G_cingul-Post-dorsal | Fi | ctx_ S_front_inf |
| Cv | ctx_ G_cingul-Post-ventral | fm | ctx_ S_front_middle |
| CN | ctx _G_cuneus | fs | ctx_ S_front_sup |
| OP | ctx_ G_front_inf-Opercular | PJ | ctx_ S_interm_prim-Jensen |
| IO | ctx_ G_front_inf-Orbital | IP | ctx_ S_intrapariet_and_P_trans |
| TR | ctx_ G_front_inf-Triangul | OL | ctx_ S_oc_middle_and_Lunatus |
| Fm | ctx _G_front_middle | ST | ctx_ S_oc_sup_and_transversal |
| Fs | ctx_ G_front_sup | oa | ctx_ S_occipital_ant |
| in | ctx _G_Ins_lg_and_S_cent_ins | Cl | ctx_S_oc-temp_lat |
| is | ctx_ G_insular_short | tl | ctx_ S_oc-temp_med_and_Lingual |
| om | ctx_ G_occipital_middle | Ol | ctx _S_orbital_lateral |
| os | ctx_ G_occipital_sup | OF | ctx_ S_orbital_med-olfact |
| TF | ctx_ G_oc-temp_lat-fusifor | HS | ctx_ S_orbital-H_Shaped |
| TL | ctx_ G_oc-temp_med-Lingual | PO | ctx_ S_parieto_occipital |
| OB | ctx_G_orbital | PE | ctx_ S_pericallosal |
| Pa | ctx_G_pariet_inf-Angular | pC | ctx_ S_postcentral |
| PS | ctx_G_pariet_inf-Supramar | ci | ctx_ S_precentral-inf-part |
| Ps | ctx_G_parietal_sup | Cs | ctx_ S_precentral-sup-part |
| Pp | ctx_ G_postcentral | sO | ctx_ S_suborbital |
| Pc | ctx_ G_precentral | sP | ctx _S_subparietal |
| Pu | ctx_ G_precuneus | ti | ctx_ S_temporal_inf |
| RT | ctx_ G_rectus | Ts | ctx_ S_temporal_sup |
| sC | ctx_ G_subcallosal | tt | ctx_ S_temporal_transverse |
| TT | ctx _G_temp_sup-G_T_transv |  |  |

Table S1: labels and associated ROI names in the Destrieux atlas ([Fischl, van der Kouwe et al. 2004](#_ENREF_4)) used for the networks of healthy subjects and simulated lesions. An additional R/L at the beginning of the label is added to indicate left and right hemispheres.
