# Supplementary material for: Validation of Network Communicability Metrics for the Analysis of Brain Structural Networks
Source: PLoS One. 2014 Dec 30;9(12):e115503. doi: 10.1371/journal.pone.0115503 (PMC4280193; doi:10.1371/journal.pone.0115503)
Supplement: S2 Table — Labels and associated ROI names in the Desikan atlas. (DOCX) [file pone.0115503.s004.docx]

| **ROI label** | **ROI name** | **ROI label** | **ROI name** |
| --- | --- | --- | --- |
| Ceb | Cerebellum Cortex | mTe | Ctx Middletemporal |
| Th | Thalamus Proper | paHip | Ctx Parahipppocampal |
| Cau | Caudate | paCe | Ctx Paracentral |
| Put | Putamen | paOp | Ctx ParsOpercularis |
| Pal | Pallidum | paOb | Ctx Parsorbitalis |
| Hip | Hippocampus | paTri | Ctx Parstriangularis |
| Amy | Amygdala | peCa | Ctx Pericalcarine |
| AccA | Accumbens Area | pCe | Ctx Postcentral |
| Bnk | Ctx Bankssts | pCin | Ctx Posteriorcingulate |
| aCin | Ctx Caudalanteriorcingulate | preCe | Ctx Precentral |
| mFr | Ctx Caudalmiddlefrontal | pCu | Ctx Precuneus |
| Cun | Ctx Cuneus | rCin | Ctx Rostralanteriorcingulate |
| Ent | Ctx Enthorinal | rFr | Ctx Rostralmiddlefrontal |
| Fus | Ctx Fusiform | sFr | Ctx Superiorfrontal |
| iPa | Ctx Inferiorparietal | sPa | Ctx Superiorparietal |
| iTe | Ctx Inferiortemporal | sTe | Ctx Superiortemporal |
| iCin | Ctx Isthmuscingulate | sMar | Ctx Supramarginal |
| lOcc | Ctx Lateraloccipital | poFr | Ctx Frontalpole |
| lOb | Ctx Lateralorbitofrontal | poTe | Ctx Temporalpole |
| Lin | Ctx Lingual | trTe | Ctx Transversetemporal |
| mOb | MedialOrbitofrontal | Ins | Ctx Insula |

Table S2: Labels and associated ROI names in the Desikan atlas ([Desikan, Segonne et al. 2006](#_ENREF_2)) used for stroke patients and matched controls subjects. An additional R/L at the beginning of the label is added to indicate left and right hemispheres.
