# Supplementary material for: Validation of Network Communicability Metrics for the Analysis of Brain Structural Networks
Source: PLoS One. 2014 Dec 30;9(12):e115503. doi: 10.1371/journal.pone.0115503 (PMC4280193; doi:10.1371/journal.pone.0115503)
Supplement: S3 Table — Hubs in the average network. (DOCX) [file pone.0115503.s005.docx]

| **Metric** | **Deg** | **S^w^** | **Cm** | **Cm^w^** |
| --- | --- | --- | --- | --- |
| **Nodes RH** | Thalamus  Caudate  Putamen  Pallidum  Frontal sup  Parietal sup  Precuneus  Pericallosal | Thalamus  Caudate  Putamen  Pallidum  Accumbens area  Insular_lg  Insular short  Circ Insula inf | Thalamus  Caudate  Putamen  Pallidum  Paracentral  Frontal middle  Frontal sup  Parietal sup  Precuneus  Precentral | Thalamus  Putamen  Accumbens area  Insular lg  Insular short  Precentral  Pericallosal |
| **Nodes LH** | Thalamus  Caudate  Putamen  Pallidum  Frontal sup  Precuneus  Pericallosal | Thalamus  Caudate  Putamen  Pallidum  Accumbens area  Insular_lg  Insular short  Plan polare  Circ Insula inf  Circ insula sup | Thalamus  Caudate  Putamen  Pallidum  Paracentral  Frontal sup  Precuneus | Thalamus  Putamen  Accumbens area  Front middle  Insular short  Occipital middle  Precuneus  Pericallosal |

Table S3: analysis of nodes with highest Deg, S^W^, Cm, Cm^W^ for the average network. We report nodes with values at least one standard deviation over the mean value. Nodes are separated into right hemisphere (RH) and left hemisphere (LH).
