# Supplementary material for: Validation of Network Communicability Metrics for the Analysis of Brain Structural Networks
Source: PLoS One. 2014 Dec 30;9(12):e115503. doi: 10.1371/journal.pone.0115503 (PMC4280193; doi:10.1371/journal.pone.0115503)
Supplement: S1 Text — Data processing and brain structural network construction. (DOCX) [file pone.0115503.s006.docx]

Text S1: Data processing and brain structural network construction

1. Motion and eddy currents correction of diffusion weighted (DW) images was performed in the Functional Magnetic Resonance Imaging of the Brain FMRIB software library version 4.1 (FSL, [http://www.fmrib.ox.ac.uk/fsl], [Smith, Jenkinson et al. (2004](#_ENREF_8))). The automated parcellation of the T1-weighted images was performed in FreeSurfer (Athinoula A. Martinos Center for Biomedical Imaging, Harvard-MIT, Boston [http://surfer.nmr.mgh.harvard.edu]). Subsequently, T1-weighted images were co-registered to the first b0 image by means of the between modality coregistration methodology using information theory, and finally re-sampled to the b0 images space. The T1-b0 transformation was also applied to atlas image using nearest neighbor interpolation. The Normalized Mutual Information cost function was employed to estimate a 12-parameter (degree of freedom) affine transformation matrix to transform voxels from MRI to b0 space. SPM5 tools (<http://www.fil.ion.ucl.ac.uk/spm5/>) were used to perform non-linear registration. For the analysis of simulated lesions, the Destrieux atlas was used, giving 154 structures that were then used as ROIs for fiber tracking ([Fischl, van der Kouwe et al. 2004](#_ENREF_4)). For the analysis of stroke patients versus controls, the lower resolution of Desikan atlas was used giving networks with 86 nodes ([Desikan, Segonne et al. 2006](#_ENREF_2)). Labels and names of the ROIs can be found in Supplementary Tables S1 and S2.
2. Probabilistic fiber tracking was performed in FSL according to [Behrens, Woolrich et al. (2003](#_ENREF_1)). A separate connectivity map was created for each ROI with seeds in each voxel of the region. Tracking parameters used were 5000 generated paths from each seed point, 0.5 mm step size, 500 mm maximum trace length and ±80 degrees curvature threshold.
3. A network is given by a set of nodes connected by edges that can be undirected or directed, weighted or unweighted. It can be represented by the adjacency matrix A in which each column/row is associated to a node and the element A_ij_>0, if there exists an edge between node i and node j. The weighted individual networks for each subject were constructed as follows:

1) Each ROI was a node.

2) An undirected edge a_ij_ between nodes i and j was established if the sum of the connectivity values between voxels of nodes i and j (or viceversa) was higher than threshold Tc (see S2.2).

3) Two edges weights w(a_ij_) were considered. For the comparison of stroke patients against controls, the count of streamlines between the voxels of nodes i and j was used (weight ACN). For the analysis of simulated lesions, the weight ACN was additionally corrected by size of nodes i and j (weight ACD).

*S1.1 The average network*

The average network was constructed using all the individual networks together. In the average network, an edge between i and j exists, if it exists in at least T_avg_=75% of the subjects. The weight of the edge is the average of the weights in the individual networks in which the edge exists ([van den Heuvel and Sporns 2011](#_ENREF_10)).

*S1.2 Connectivity and density thresholds*

Two different types of threshold were set on the individual weighted networks. First, a connectivity threshold (Tc) was set in order to eliminate low connection probabilities. This threshold was adjusted for the two different atlases and weights. For the Destrieux atlas, Tc=0.0005, while for the Desikan atlas the threshold was set to Tc=3.8x10^4^. The threshold used for the analysis of stroke patients was more severe, because tractography may be more difficult in this case.

In a supplementary analysis, an additional threshold TD on the density (i.e. the proportion of existing edges over the total number of possible edges) was used in order to obtain networks with the same number of edges and nodes. The density threshold TD was set to the maximum common density over all subjects.
