# Supplementary material for: Validation of Network Communicability Metrics for the Analysis of Brain Structural Networks
Source: PLoS One. 2014 Dec 30;9(12):e115503. doi: 10.1371/journal.pone.0115503 (PMC4280193; doi:10.1371/journal.pone.0115503)
Supplement: S2 Text — Graph metrics definitions. (DOCX) [file pone.0115503.s007.docx]

Text S2: Graph metrics definitions

In the section here after the standard graph metrics considered in our analysis are defined: degree, strength, distance measures, efficiency and betweenness centrality. We denote A^w^ the weighted adjacency matrix of the network and A the binary connectivity matrix. The notation ^w^ indicates weighted network metrics, i.e. metrics computed using W. Properties characterizing the whole network organization are denoted as global, while local properties are specific for each node. A more detailed description of network properties and their interpretations can be found in [Rubinov and Sporns (2010](#_ENREF_7)).

*S3.1 Degree and strength*

The degree of a node (Deg) is the number of edges connected to it. The global degree of the network is the average degree over all nodes. Hubs were defined as nodes with degree at least one standard deviation over the mean degree ([Sporns, Honey et al. 2007](#_ENREF_9)). Similarly, in the weighted case, the strength of a node (S^w^) is given by the column sum of W.

*S3.2 Distance measures and characteristic path length*

The length of every edge in a network is given by the inverse of its weight. In binary networks each edge has length 1. The distance function of a network gives the length of the shortest path between any two nodes and can be computed using the Dijikstra algorithm ([Dijkstra 1959](#_ENREF_3)). The average shortest path length (L) over all pairs of nodes is called characteristic path length ([Watts and Strogatz 1998](#_ENREF_11)). In addition, the distance matrix was used and denoted Dist.

*S3.3 Efficiency*

The global efficiency (Eff) is a measure of the functional integration in the network, which is the ability of fast communication also between distributed regions ([Latora and Marchiori 2001](#_ENREF_6)). Global efficiency is the average of node’s efficiency computed as

${Eff}_{i}=\frac{1}{N}\sum_{j} \frac{Dist_{ij}^{-1}}{N-1}$,

where $dist_{ij}$ is the length of the shortest path between i and j (computed for A or W) and N is the total number of nodes .

*S3.4 Betweenness centrality*

The betweenness centrality (BC) of a node is the number of shortest paths in the network that pass through it. It can be normalized over the total number of shortest paths. A large flow of information will pass through the shortest paths and hence through nodes with high BC ([Freeman 1979](#_ENREF_5)).
