# Supplementary material for: Validation of Network Communicability Metrics for the Analysis of Brain Structural Networks
Source: PLoS One. 2014 Dec 30;9(12):e115503. doi: 10.1371/journal.pone.0115503 (PMC4280193; doi:10.1371/journal.pone.0115503)
Supplement: S3 Text — Group comparison at baseline. (DOCX) [file pone.0115503.s008.docx]

Text S3. Group comparisons at baseline

In order to model the situation of longitudinal measurements, for each subject two different consecutives measurements were acquired and we separate the networks obtained randomly into two groups (one measurement per patient in each group). The first set of measurements was then used as "baseline" situation, while the second set was used to simulate the progression of a disease by applying different types of lesions.

First we tested for differences between the two groups.

| **Global Metrics** | **p-values** | | |  | **p-values** | | |
| --- | --- | --- | --- | --- | --- | --- | --- |
| **Binary** | **Whole** | **Right** | **Left** | **Weighted** | **Whole** | **Right** | **Left** |
| **Degree** | 0.52 | 0.44 | 0.61 | **S** | 0.98 | 0.98 | 0.87 |
| **BC** | 0.94 | 0.82 | 0.92 | **BC^w^** | 0.07 | **<0.05** | 0.71 |
| **Cm** | 0.22 | 0.98 | 0.33 | **Cm^w^** | 0.56 | 0.93 | 0.27 |
| **CBC** | 0.62 | 0.86 | 0.82 | **CBC^w^** | 0.49 | 0.70 | 0.26 |

Table S5.1: comparison of network metrics at baseline (between measurements).

Differences are not significant (ns), except for BC^w^ in the right hemisphere. This result will be considered in the analysis.

| **Local metrics - Bin** | **N of nodes** | **Corrected FDR** | **Weighted** | **N of nodes** | **Corrected FDR** |
| --- | --- | --- | --- | --- | --- |
| **Degree** | 7 | 0 (ns) | **S** | 0 | 0 (ns) |
| **BC** | 8 | 0 (ns) | **BC^w^** | 1 | 0 (ns) |
| **Cm** | 5 | 0 (ns) | **Cm^w^** | 10 | 0 (ns) |
| **CBC** | 7 | 0 (ns) | **CBC^w^** | 7 | 0 (ns) |

Table S5.2: comparison of local metrics at baseline (between measurements).

After correction differences are not significant. The False Discovery Rate was applied for correction for multiple testing (Benjamini and Yekutieli, 2001).
