# Supplementary material for: Validation of Network Communicability Metrics for the Analysis of Brain Structural Networks
Source: PLoS One. 2014 Dec 30;9(12):e115503. doi: 10.1371/journal.pone.0115503 (PMC4280193; doi:10.1371/journal.pone.0115503)
Supplement: S4 Text — Analysis of the effects of variability in 150 of random attacks to nodes. (DOCX) [file pone.0115503.s009.docx]

Text S4. Analysis of the variability across 150 repetitions of random binary lesions to nodes

| **Random attacks** | | **R=0.2,**  **same nodes** | | | | **R=0.5,**  **same nodes** | | | | **R=0.8,**  **same nodes** | | | | **R=0.8,**  **individual nodes** | | | |
| --- | --- | --- | --- | --- | --- | --- | --- | --- | --- | --- | --- | --- | --- | --- | --- | --- | --- |
| **Number** | | **3** | **6** | **9** | **12** | **3** | **6** | **9** | **12** | **3** | **6** | **9** | **12** | **3** | **6** | **9** | **12** |
| **Deg** | Mean | 0.2 | 3.9 | 7.3 | 10.2 | 2.9 | 6.0 | 10.2 | 17.7 | 3.0 | 8.7 | 22.8 | 38.5 | 0.0 | 0.0 | 5.6 | 37.7 |
|  | SD | 0.7 | 2.1 | 1.6 | 1.4 | 0.5 | 0.6 | 2.0 | 4.3 | 0.5 | 3.9 | 8.1 | 10.1 | 0.0 | 0.0 | 7.8 | 7.4 |
| **S** | Mean | 0.0 | 0.1 | 0.4 | 1.5 | 1.3 | 6.1 | 10.4 | 15.4 | 4.7 | 11.8 | 19.8 | 28.2 | 0.0 | 0.0 | 0.0 | 1.7 |
|  | SD | 0.0 | 0.4 | 1.2 | 2.5 | 1.6 | 1.5 | 2.1 | 2.7 | 1.7 | 2.8 | 3.4 | 4.3 | 0.0 | 0.0 | 0.0 | 4.0 |
| **BC** | Mean | 0.0 | 0.0 | 0.0 | 0.1 | 0.7 | 4.1 | 7.2 | 11.3 | 2.8 | 7.1 | 10.1 | 15.8 | 0.0 | 0.0 | 0.0 | 0.0 |
|  | SD | 0.0 | 0.0 | 0.0 | 0.6 | 1.3 | 2.5 | 3.7 | 6.4 | 1.8 | 5.0 | 7.5 | 9.6 | 0.0 | 0.0 | 0.0 | 0.0 |
| **WBC** | Mean | 0.0 | 0.0 | 0.0 | 0.0 | 0.0 | 0.1 | 0.4 | 0.7 | 1.1 | 5.7 | 10.6 | 15.3 | 0.0 | 0.0 | 0.0 | 0.0 |
|  | SD | 0.0 | 0.0 | 0.0 | 0.0 | 0.2 | 0.7 | 1.2 | 2.0 | 1.7 | 3.6 | 3.7 | 4.8 | 0.0 | 0.0 | 0.0 | 0.2 |
| **Cm** | Mean | 0.5 | 3.5 | 6.5 | 9.1 | 2.9 | 6.0 | 9.2 | 12.5 | 2.9 | 6.1 | 9.6 | 13.1 | 0.0 | 0.0 | 0.0 | 0.0 |
|  | SD | 1.1 | 2.1 | 1.9 | 1.8 | 0.5 | 0.6 | 1.2 | 2.1 | 0.5 | 1.1 | 2.3 | 3.3 | 0.0 | 0.0 | 0.0 | 0.0 |
| **CmW** | Mean | 0.0 | 0.3 | 1.2 | 2.0 | 2.1 | 6.5 | 11.1 | 15.8 | 4.2 | 11.1 | 18.5 | 25.2 | 0.0 | 0.0 | 0.0 | 0.0 |
|  | SD | 0.0 | 1.1 | 2.2 | 2.8 | 1.5 | 1.6 | 2.6 | 3.9 | 2.0 | 4.5 | 5.5 | 6.3 | 0.0 | 0.0 | 0.0 | 0.0 |
| **CBC** | Mean | 0.2 | 2.6 | 7.8 | 14.5 | 3.3 | 14.0 | 31.0 | 52.1 | 6.8 | 22.3 | 46.0 | 70.9 | 0.0 | 1.4 | 29.9 | 71.6 |
|  | SD | 0.8 | 2.5 | 4.4 | 7.0 | 2.4 | 16.3 | 24.2 | 28.0 | 12.0 | 26.7 | 31.1 | 28.5 | 0.0 | 3.5 | 16.2 | 13.3 |
| **CBCw** | Mean | 0.0 | 0.0 | 0.2 | 0.4 | 1.7 | 5.6 | 9.2 | 12.7 | 3.7 | 8.7 | 14.0 | 19.3 | 0.0 | 0.0 | 0.0 | 0.0 |
|  | SD | 0.0 | 0.3 | 0.7 | 1.1 | 1.6 | 1.6 | 2.6 | 3.7 | 2.8 | 5.8 | 7.8 | 9.1 | 0.0 | 0.0 | 0.0 | 0.0 |

Table S4.1: Average number (mean) and variability (SD) of significant local changes over 150 repetitions for the various metrics when random nodes were targeted for binary lesions.


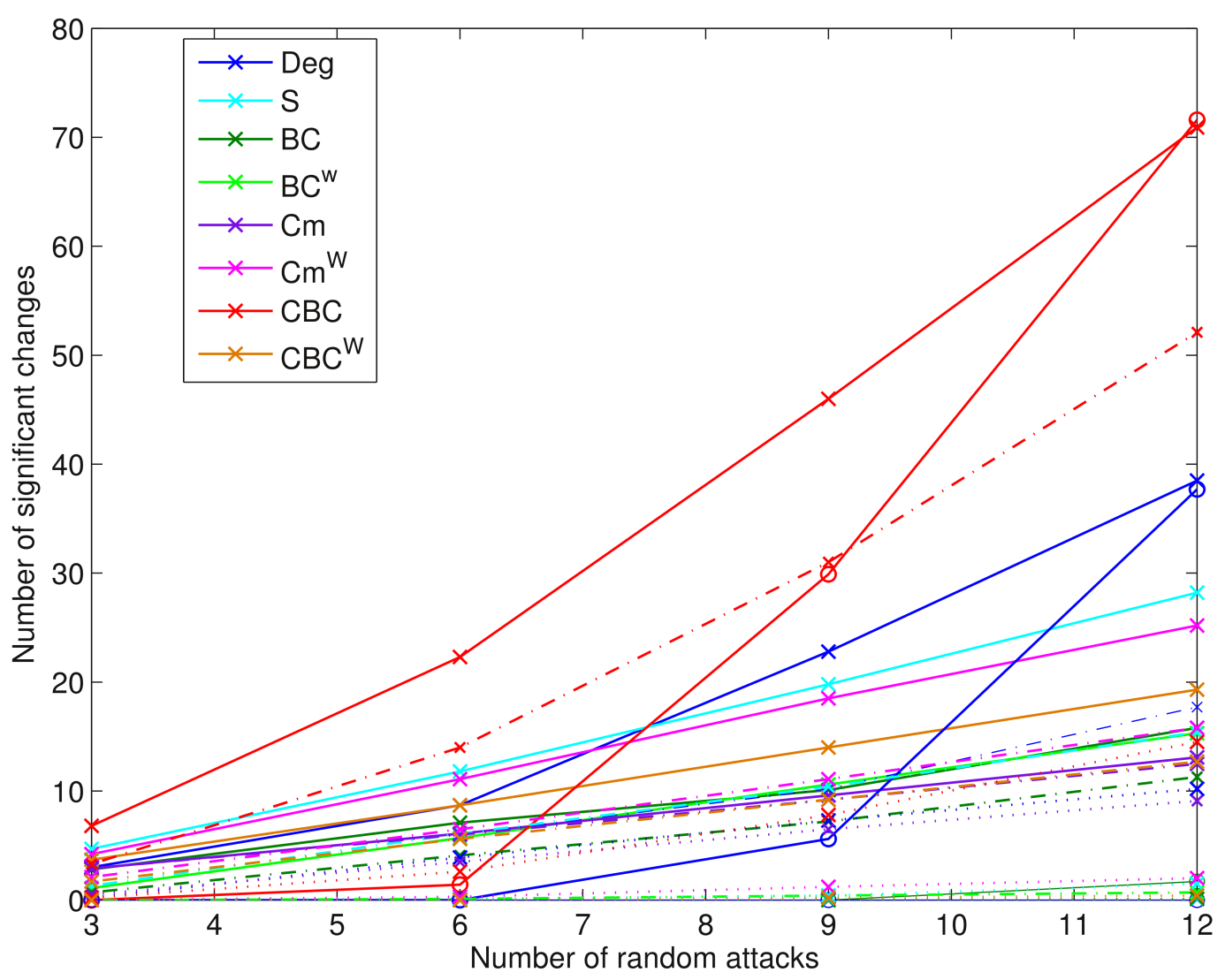


Figure S4.1: average number of significant local changes over 150 repetitions for the various metrics when random nodes were targeted for binary lesions. The line types indicate the site selection and rate: solid lines with cross markers for same nodes for all subjects and rate R=0.8, dashed lines with cross markers for same nodes for all subjects and R=0.5, dotted lines for with cross markers for same nodes for all subjects and R=0.2 and solid lines with circle markers different nodes for each subject R=0.8.
