# Supplementary material for: Validation of Network Communicability Metrics for the Analysis of Brain Structural Networks
Source: PLoS One. 2014 Dec 30;9(12):e115503. doi: 10.1371/journal.pone.0115503 (PMC4280193; doi:10.1371/journal.pone.0115503)
Supplement: S5 Text — Analysis of the distributions of local changes. (DOCX) [file pone.0115503.s010.docx]

Text S5. Analysis of the distributions of local changes

In Table S5.1, we report the individual and average correlation coefficients between local changes and distance from lesions in the case of random lesions. The lesion site is excluded in this computation. Notice that the correlations are generally less strong then in the case of attacks to hubs. In particular, in individual lesions the standard deviation is high, probably due to the reduced magnitude of changes as compared to lesions to hubs. However, in line with the case of lesions to hubs, in most of the cases the correlations become stronger when more nodes are damaged.

| **Number of attacks** | | **1** | | **5** | | **9** | |
| --- | --- | --- | --- | --- | --- | --- | --- |
| **Correlation** | | **Individual** | **Average** | **Individual** | **Average** | **Individual** | **Average** |
| **Deg** | DistW | -0.000±0.11 | -0.08 | -0.08±0.14 | -0.27 | -0.13±0.14 | -0.35 |
| **S^w^** | DistW | 0.02±0.11 | -0.09 | -0.09±0.13 | -0.29 | -0.14±0.11 | -0.35 |
| **BC** | DistW | 0.03±0.04 | 0.12 | 0.06±0.05 | 0.22 | 0.08±0.04 | 0.25 |
| **BC^w^** | DistW | 0.04±0.04 | 0.08 | 0.04±0.04 | 0.08 | 0.03±0.04 | 0.07 |
| **Cm** | DistW | 0.04±0.06 | 0.11 | 0.05±0.07 | 0.16 | 0.05±0.07 | 0.15 |
| **Cm^w^** | DistW | 0.02±0.05 | -0.04 | -0.00±0.04 | -0.06 | -0.03±0.04 | -0.09 |
| **CBC** | DistW | -0.02±0.19 | -0.14 | -0.13±0.21 | -0.38 | -0.21±0.16 | -0.49 |
| **CBC^w^** | DistW | 0.04±0.05 | 0.10 | 0.08±0.05 | 0.23 | 0.10±0.05 | 0.28 |

Table S5.1: Correlation coefficients between local metric changes and (weighted and binary) distance from lesions in the case of random lesions. Mean and standard deviations of individual correlations are reported (Individual) as well as the correlation coefficients between the average change and the average distance from lesions (Average).

In addition, we analyzed the location of the most significant changes for all types of binary lesions in order to understand if some regions were more sensitive to the lesion in general.

For hubs lesions, the most significant changes were defined based on the corrected p-values after 5 attacks, i.e. the sites with lower p-values where considered to be the most significant changes. For random nodes and edges lesions, the most significant changes were selected based on the frequency of a significant change in a specific region (after 5 attacks and 10 repetitions).

The most significant changes are reported in Figure S5.1, S5.2 and S5.3 for hubs, random nodes and random edges lesions respectively. Additionally, a summary of the regions that are more frequently found is given in Table S5.2.


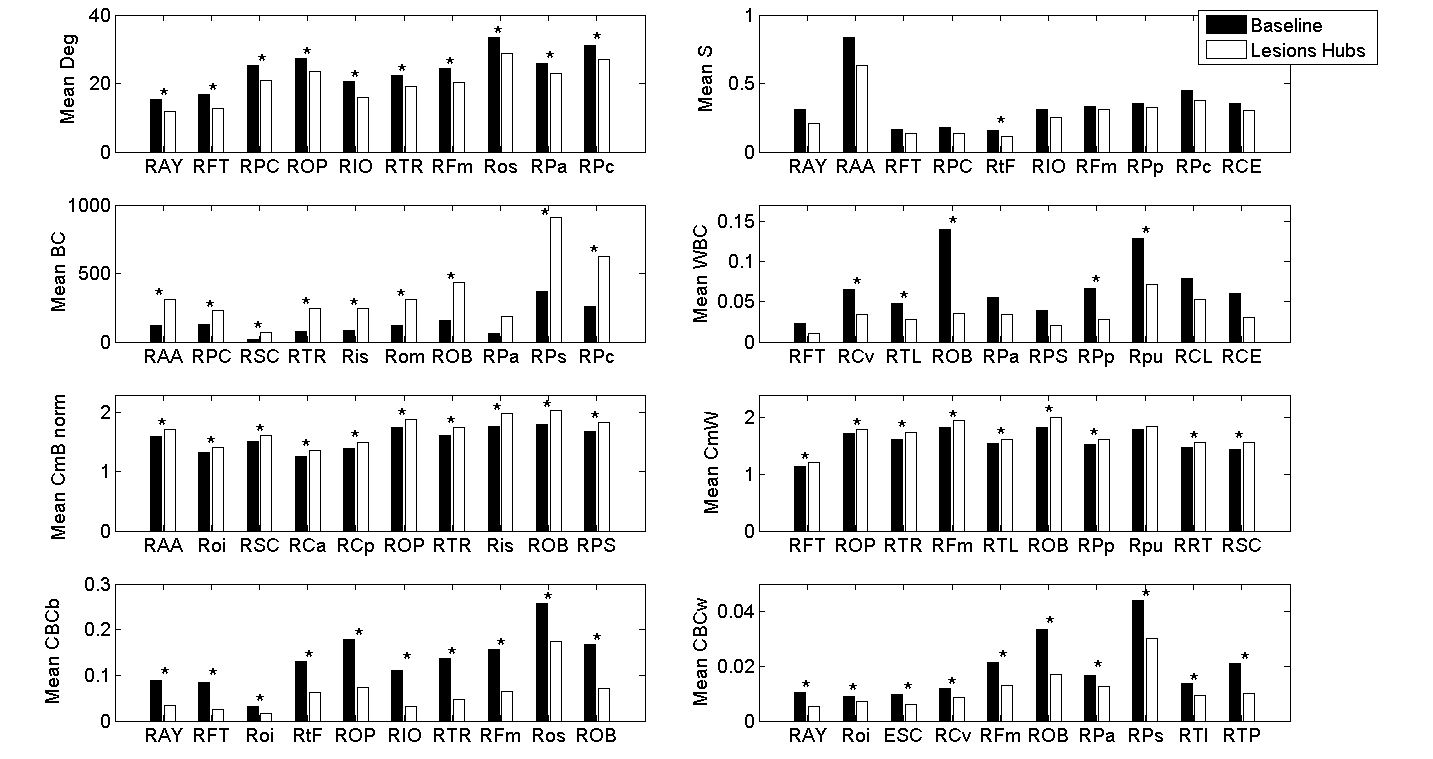


Figure S5.1: most significant changes after 5 attacks to hubs nodes (excluding the lesion sites). * indicate significant changes. Black bars are for local metrics at baseline and white bars are for metrics after 5 attacks to hubs nodes.


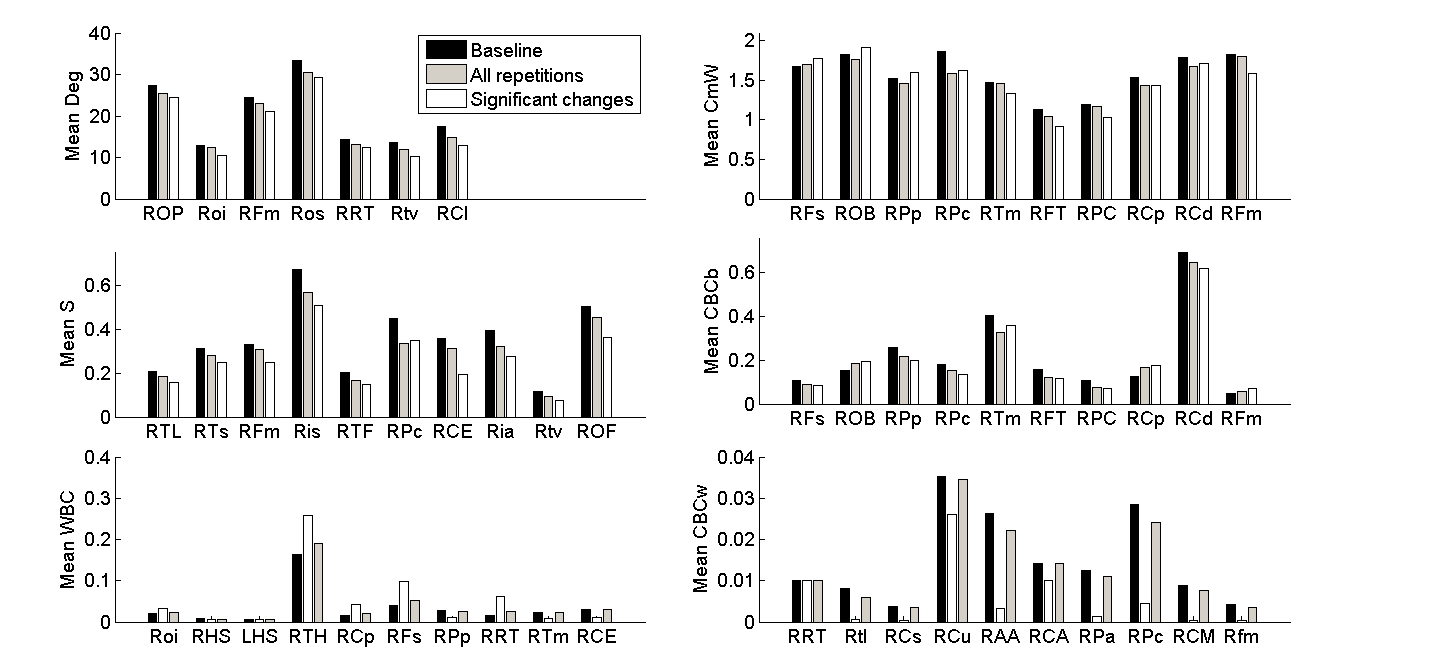


Figure S5.2: most significant changes after 5 attacks to random nodes (excluding the lesion sites). Black bars are for local metrics at baseline, gray bars are for metrics after 5 attacks averaged over all repetitions, white bars are for metrics after 5 attacks averaged over repetitions where the nodes had a significant change.


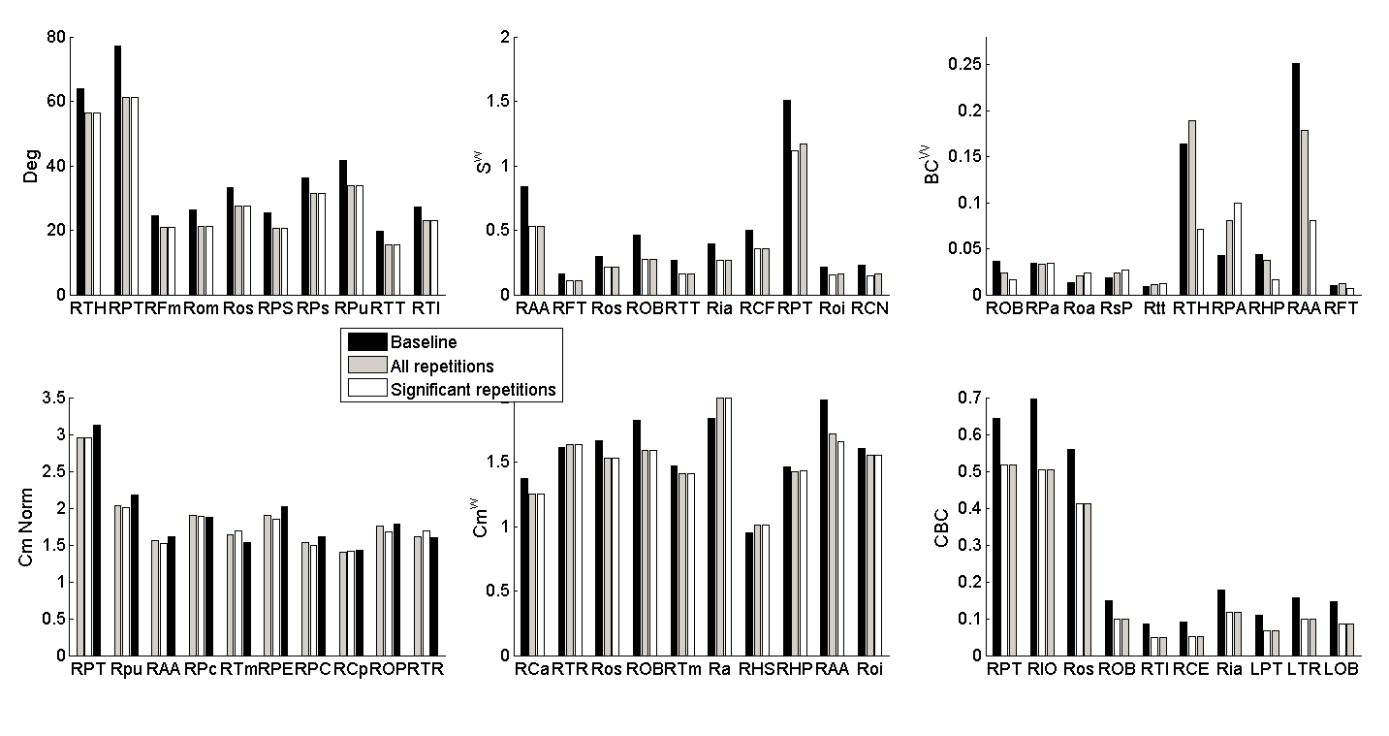


Figure S5.3: most significant changes after attacks to 150 random edges. Black bars are for local metrics at baseline, gray bars are for metrics after the attacks averaged over all repetitions, white bars are for metrics after the attacks averaged over repetitions where the nodes had a significant change.

|  | **Overall** | **All Hubs** | **Random** | **All Edges** | **Standard** | **Communicability** |
| --- | --- | --- | --- | --- | --- | --- |
| **RAA** | 9 | 3 | 1 | 5 | 4 | 5 |
| **RAY** | 4 | 4 | 0 | 0 | 2 | 2 |
| **RFT** | 8 | 5 | 1 | 2 | 5 | 3 |
| **RFm** | 11 | 5 | 4 | 2 | 6 | 5 |
| **RPc** | 10 | 3 | 3 | 4 | 6 | 4 |
| **Rpu** | 8 | 2 | 1 | 5 | 4 | 2 |
| **RPC** | 6 | 3 | 1 | 2 | 4 | 2 |
| **Ria** | 4 | 0 | 1 | 0 | 2 | 2 |
| **Ris** | 4 | 0 | 1 | 1 | 3 | 1 |
| **ROB** | 14 | 6 | 2 | 6 | 4 | 10 |
| **Ros** | 10 | 2 | 2 | 6 | 3 | 1 |
| **RPA** | 4 | 0 | 0 | 4 | 3 | 1 |
| **RPa** | 8 | 4 | 1 | 3 | 6 | 2 |
|  |  |  |  |  |  |  |

Table S5.2: frequency of nodes detected as most significant over all metrics and all types of binary lesions (in total 4 types of lesions and 8 metrics were considered).
