# Supplementary material for: Validation of Network Communicability Metrics for the Analysis of Brain Structural Networks
Source: PLoS One. 2014 Dec 30;9(12):e115503. doi: 10.1371/journal.pone.0115503 (PMC4280193; doi:10.1371/journal.pone.0115503)
Supplement: S6 Text — Analysis of simulated stroke lesions and stroke patients. (DOCX) [file pone.0115503.s011.docx]

Text S6. Analysis of simulated stroke lesions and stroke patients

|  | **Deg** | **S^w^** | **BC** | **BC^w^** |
| --- | --- | --- | --- | --- |
| **R** | Precuneus |  | G Rectus |  |
| **L** | Thalamus  Caudate | Thalamus  Caudate  Hippocampus  Accumbens Area | Thalamus  Caudate  Putamen  G subcallosal  S orbital lateral | Thalamus |
|  | **Cm** | **Cm^w^** | **CBC** | **CBC^w^** |
| **R** | G +S cingulate mid anterior | G Front superior | G+S subcentral  Circular insula |  |
| **L** | Thalamus  Caudate | Thalamus  Caudate  Hippocampus  G orbital  G postcentral  G rectus | Thalamus  Caudate  G orbital | Thalamus  Caudate  Amygdala  Hippocampus  S orbital med-olfact |

Table S6.1: Summary of significant changes in the average network over all simulated stroke lesions (applied to the left thalamus and caudate nuclei).


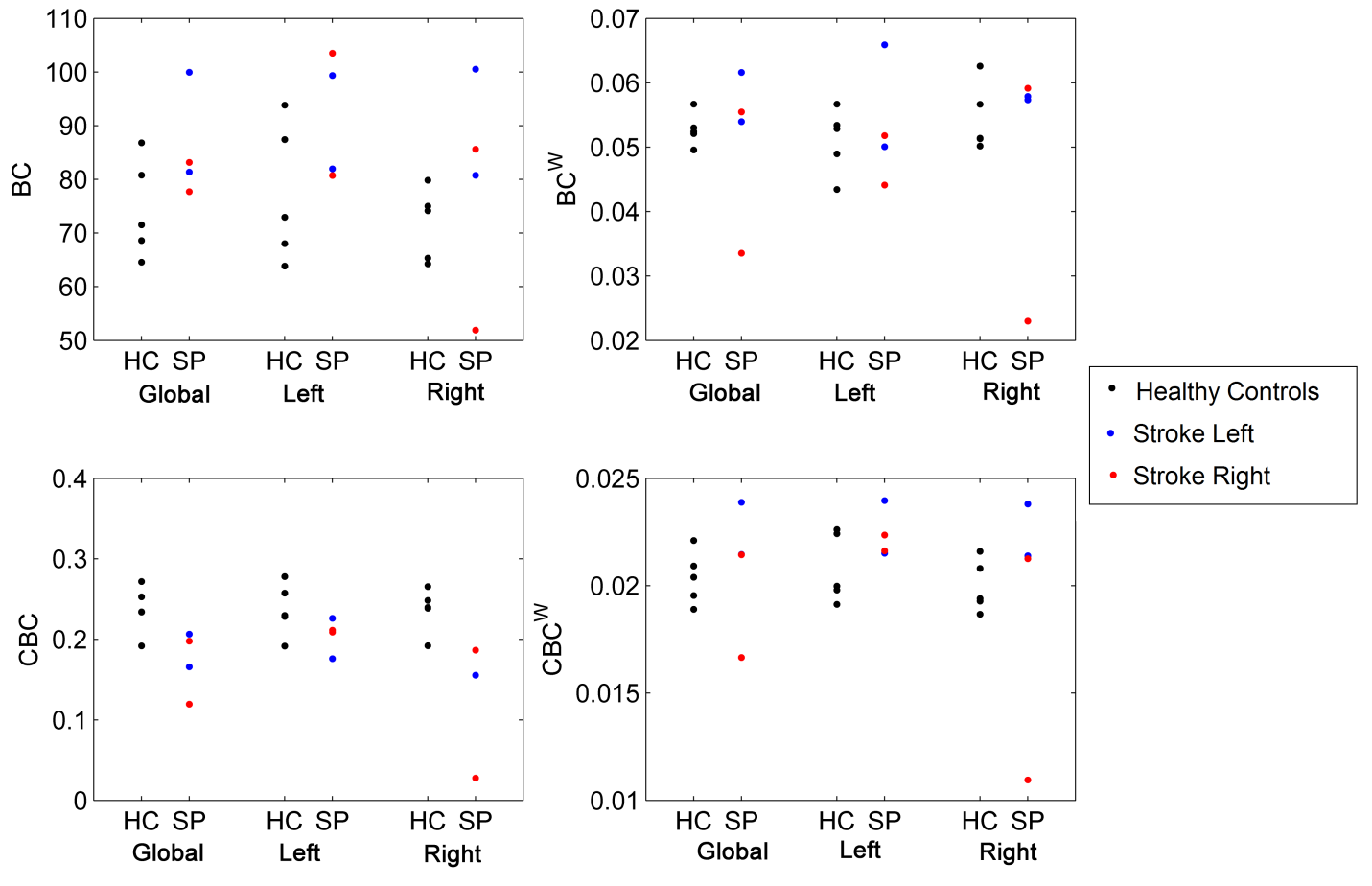


Figure S6.1: global and hemispheric network metrics of BC, CBC, BC^w^ and CBC^w^ for healthy controls (HC) against stroke patients (SP).
